# The interplay between cognitive and psychological factors in subjective cognitive decline: contribution to the validation of a new screening battery

**DOI:** 10.3389/fpsyg.2025.1670551

**Published:** 2025-10-09

**Authors:** Marina Maffoni, Annalisa Magnani, Antonia Pierobon, Alessandra Mafferra, Fabrizio Pasotti, Carlo Dallocchio, Pierluigi Chimento, Valeria Torlaschi, Giuseppe Trifirò, Cira Fundarò

**Affiliations:** ^1^Psychology Unit, Istituti Clinici Scientifici Maugeri IRCCS, Montescano Institute, Montescano, Italy; ^2^Department of Psychology, Università Cattolica del Sacro Cuore, Milan, Italy; ^3^Neurology Unit, Department of Medical Area, ASST di Pavia, Pavia, Italy; ^4^Department of Rehabilitation ASST Pavia, Voghera, Italy; ^5^Neurophysiopathology Unit, Istituti Clinici Scientifici Maugeri IRCCS, Montescano Institute, Italy, Montescano, Italy; ^6^Istituti Clinici Scientifici Maugeri IRCCS, Nuclear Medicine Unit of Pavia Institute, Pavia, Italy

**Keywords:** subjective cognitive decline, depressive symptoms, preventive interventions, neuropsychological evaluation, assessment, rehabilitation

## Abstract

**Background:**

Subjective Cognitive Decline (SCD) is increasingly recognized as a potential early indicator of neurodegenerative disorders, yet its heterogeneous nature and lack of standardized screening tools complicate early detection and clinical management. This study aimed to comprehensively characterize a clinical SCD population and provide a preliminary contribution to the validation of a novel multidimensional screening battery, called MASCoD (Multidimensional Assessment of Subjective Cognitive Decline).

**Methods:**

A total of 59 individuals (69.36 ± 8.66, female: 71,2%) with self-reported SCD without objective cognitive impairment was recruited within two Centers for Cognitive Disorders and Dementia (CCDDs) in Northern Italy. Participants underwent a comprehensive assessment, including neurological assessment, neuropsychological testing, psychological screening, and administration of MASCoD. Convergent validity was assessed using the Cognitive Function Instrument (CFI), and internal consistency was evaluated too. Correlations between MASCoD subscales, depressive and anxious symptoms, and cognitive performance were examined, alongside comparisons between short and long forms of anxiety and depression measures (GAD and PHQ).

**Results:**

The sample showed preserved cognitive performance across all domains, consistent with SCD diagnostic criteria. Mild depressive symptoms were present and significantly associated with SCD, explaining up to around 10% of the variance in SCD measures. MASCoD Section B demonstrated good internal consistency (KR-20 = 0.782) and the entire tool shows moderate-to-strong convergent validity with the CFI. Notably, comparisons between PHQ-2/GAD-2 and their full-length versions indicated the brief tools may underestimate affective symptoms in this population.

**Conclusion:**

This study offers a detailed clinical and cognitive profile of an older SCD population, emphasizing the psychological dimensions of subjective complaints which should be evaluated into routine cognitive screening to proposed targeted preventive and rehabilitation interventions. The preliminary validation of MASCoD supports its potential as a reliable and multidimensional screening tool for early SCD detection. However, limitations such as small sample size, cross-sectional design, and cultural specificity necessitate further validation studies.

## Introduction

Subjective Cognitive Decline (SCD) is increasingly recognized as a clinically meaningful phenomenon in aging populations. It is defined as a self-perceived decline in cognitive abilities without corresponding objective deficits detectable through conventional neuropsychological assessments (Jessen et al., 2014, 2020). Despite the absence of measurable impairment, emerging longitudinal evidence supports the prognostic relevance of SCD. Individuals with SCD demonstrate a two- to four-fold increased risk of conversion to MCI and dementia over time compared to those without subjective complaints (Mitchell et al., 2014; Slot et al., 2019). Specifically, the SCD Initiative (SCD-I) has contributed to the conceptual refinement of this construct, proposing that subjective complaints in memory or other cognitive domains—particularly when coupled with SCD-plus features such as age of onset over 60, recent symptom emergence, and concern regarding cognitive changes—may mark the earliest symptomatic stage of AD (Jessen et al., 2014, 2020; Molinuevo et al., 2017). Thus, early identification provides an opportunity for timely intervention, potentially altering the trajectory of cognitive decline and improving patient outcomes (Jessen et al., 2020; Numbers et al., 2021).

The prevalence of SCD varies significantly across studies, with some estimates indicating that up to 50–60% of community-dwelling older adults may experience SCD (Holmen et al., 2013; Singh-Manoux et al., 2014). This variation can be attributed to differences in population characteristics, assessment methods, and cultural factors, underlining the importance of standardized, multidimensional approaches to SCD evaluation. Notably, SCD is more prevalent among individuals with lower education, lower socioeconomic status, and higher psychosocial vulnerability, emphasizing the need for context-specific assessment tools (Röhr et al., 2020). However, a clear definition of the core characteristic of the SCD population is not available. What the literature is beginning to show is that, although the results are still not all in agreement, higher education and other proxies for cognitive reserve (e.g., cognitive life activities) can delay the conversion of SCD to cognitive decline (Arora et al., 2024).

Despite its clinical importance, there is currently no single gold standard for the objective detection of SCD. Current diagnostic approaches rely on either disease-cent red or biomarker-centered guidelines according to the patient’s clinical profile, combining neurological and clinical evaluations, neuropsychological assessments, and advanced neuroimaging techniques, such as positron emission tomography (PET). Also, biomarkers - including cerebrospinal fluid analysis and neurodegeneration blood-based markers – can be used to identify early pathological changes and this is relevant for the advent of growing disease-modifying therapies (Frisoni et al., 2024). In addition, some self-reported questionnaire exists. For example, one of the earliest and still widely used instruments is the six-item Memory Complaint Questionnaire (MAC-Q; Crook et al., 1992; Reid et al., 2012), designed to detect perceived memory decline. Each item is rated on a 5-point Likert scale, where participants indicate whether they perceive their memory as better or worse compared to the past. The total score ranges from 7 (with the sixth item weighted double) to 35, and scores of 25 or higher indicate clinically significant subjective memory difficulties. Other existing tools should be mentioned. The Prospective and Retrospective Memory Questionnaire (PRMQ; Smith et al., 2000) is a 16-item self-report tool that evaluates common everyday memory lapses in both prospective (8 items) and retrospective (8 items) memory through items rated on a 5-point Likert scale from “Very often” to “Never,” with higher scores reflecting greater perceived impairment. The Memory Complaint Scale - MCS (Vale et al., 2012) consists of two versions: Version A, a 7-item self-report addressed to the patient, and Version B, a 7-item questionnaire addressed to the patient’s companion. Each item uses a three-option Likert scale (e.g., frequency or yes-no-unsure). Scores are interpreted as follows: 0–2 indicates no memory complaints, 3–6 mild complaints, 7–10 moderate complaints, and 11–14 severe complaints. Finally, the Kihon Checklist-Cognitive Function (KCL-CF; Tomata et al., 2017) is a brief screening tool designed to identify early cognitive concerns as part of a broader health assessment. Specifically, it consists of three yes (=1)/no (=0) items assessing memory complaints: (i) being reminded by others of memory loss, (ii) needing to look up phone numbers when making calls, and (iii) not knowing the current date. It is primarily used to screen high-risk older adults, with a cut-off score of ≥1 demonstrating a specificity of 65.1% and a sensitivity of 60.2% for detecting cognitive impairment.

However, most questionnaires remain limited in scope, lack translations into many languages, and often neglect non-memory domains or psychological correlates of SCD. In an attempt to bridge this gap, it is interesting that the self-reported Cognitive Function Instrument - CFI (Walsh et al., 2006; Chipi et al., 2018; Li et al., 2023) is used for monitoring annual variations in global cognitive functioning, but fails to include a specific focus on psychological symptoms too. It is available in two versions—one for the patient and one for the caregiver—each consisting of 14 items with responses scored as yes (1), unsure (0.5), or no (0). The self-report version addresses the individual’s difficulties in everyday cognitive tasks such as remembering appointments, managing finances, recalling names, or experiencing more difficulties in social activities or reading. The partner-report version captures similar observations from a close informant such as a spouse or family member. Higher scores indicate greater cognitive complaints.

Furthermore, the heterogeneity of SCD complicates early detection. It can arise from various etiologies, ranging from psychological factors, such as anxiety and depression, to the initial stages of neurodegenerative processes. Literature shows that grief, anxious and depressive symptoms may play a role in the clinical manifestation of SCD. For some individuals, complaints are driven primarily by psychological factors such as anxiety, depression, or health-related worry, which can amplify attention to minor memory lapses (Amariglio, 2021). Mood disorders are both a potential predictor and consequence of SCD and are associated with poorer outcomes over time (Hill et al., 2016; Yates et al., 2017). This bidirectional relationship underscores the importance of assessing emotional health when evaluating SCD. There is still uncertainty concerning the optimal screening instruments for depressive and anxiety symptoms. Specifically, abbreviated screening tools, such as the well-known PHQ-2 and GAD-2 (Giuliani et al., 2021), have not yet been systematically validated across all settings, leaving uncertainty regarding their concordance and diagnostic overlap with their comprehensive, full-length counterparts (Lang et al., 2009).

Specifically considering cognitive performance, clinical practice and research on SCD reveals significant challenges in establishing standardized diagnostic criteria, particularly regarding neuropsychological testing thresholds. A central challenge lies in determining whether to employ more demanding neuropsychological assessments or to adopt more stringent standardized grading scales. Pestana et al. (2024) demonstrated that SCD diagnosis frequency varies dramatically (16.4 to 81.3%) depending on the neuropsychological criteria used, with more conservative thresholds (≥2 tests >1.5 SD below norms) identifying patients with poorer global cognitive function compared to liberal criteria (1 test >1 SD below norms). This variability highlights the lack of consensus on standard score cut-offs for differentiating SCD from mild cognitive impairment. Two primary approaches have emerged to address these limitations. De Simone et al. (2023) found that more challenging experimental tasks assessing associative memory and spatial pattern separation achieved 79–82% accuracy in detecting SCD-related cognitive changes that traditional tests missed. Marra et al. (2021) suggested that the semantic–phonological delta might reflect early impairments in semantic memory among individuals at the very initial stages of Alzheimer’s disease. Additionally, Rabin et al. (2015) documented substantial heterogeneity across 34 self-report measures used internationally, with approximately 75% of measures used by only a single study, emphasizing the need for more restrictive standardized assessment approaches.

Given these complexities, a critical preliminary step is still today to better define and characterize the population with SCD, according to demographic, psychological and neuropsychological perspectives. Indeed, the diversity unveiled by research and clinical practice suggests that SCD may encompass distinct subtypes, each with unique risk profiles and clinical trajectories, highlights the need for integrated, biopsychosocial targeted diagnostic approaches. Thus, there is a pressing need for brief, cost-effective, multidimensional and psychometrically valid screening instruments that can capture the wide range of cognitive and psychological manifestations (specifically anxious and depressive symptoms as most represented in literature) associated with SCD (Ibnidris et al., 2022). The clear definitions of different SCD profiles and the development of comprehensive tools will be essential for early diagnosis, personalized preventive or rehabilitation interventions, and improved patient outcomes.

## Objectives

In line with the above-mentioned recent literature underscoring the heterogeneity of SCD and the need for reliable, multidimensional screening instruments, this study had three main objectives:

1. To characterize the sample by describing the socio-demographic profile and neuropsychological and psychological performance of individuals with SCD, thereby providing an overview of the population under study.
2. To conduct a preliminary contribution to the validation of a novel screening tool designed for the early identification and longitudinal tracking of SCD:

2a. To calculate MASCoD internal consistency reliability and convergent validity with CFI,
2b. To further explore construct validity of the MASCoD by examining its correlations with established cognitive and psychological measures, so supporting its theoretical grounding.

3. To investigate the relationship between depressive and anxiety symptoms and SCD:

3a. Due to the uncertainty surrounding the validity of short forms compared to their full-length counterparts, we aimed to evaluate the convergence between the short and full-length versions of depression and anxiety scales in this population by comparing symptom severity estimates from the PHQ-2 with the PHQ-9, and from the GAD-2 with the GAD-7, thereby providing valuable insights to optimize clinical screening strategies.
3b. To assess the predictive value of depressive symptoms on SCD outcomes.

## Method

### Study design

This study is a prospective controlled clinical design including an assessment (neurological evaluation, brain 18F-FDG-PET if clinically eligible, psychological and neuropsychological assessment) and 2-month technology-assisted cognitive training. Detailed information regarding the entire study protocol that is still in progress can be found on ClinicalTrials.gov (Identifier: NCT05815329).

Here, we present only the initial evaluation (T0), focusing specifically on clinical, neurological, and neuropsychological assessments to both help characterize the profile of the SCD population (aim 1) and preliminarily contribute to the validation of a new screening tool for SCD called MASCoD, which has been described elsewhere (Maffoni et al., 2022) (aim 2).

### Procedure

Participants first underwent a neurological consultation as part of routine clinical practice within the Centers for Cognitive Disorders and Dementia (CCDDs) in Italy (Bacigalupo et al., 2024). All clinical activities, including diagnostics, instrumental examinations, rehabilitation interventions, and patient care, are standardized within recruiting centers according to internal procedures (Diagnostic Therapeutic Care Pathway – DTCP), which are aligned with international guidelines for the management of cognitive disorders (Frisoni et al., 2024). In detail, if the neurologist identified symptoms potentially consistent with SCD and no prior diagnosis of cognitive impairment was present, patients were invited to participate in the study. Upon receiving detailed information about the research protocol, eligible individuals provided written informed consent. Subsequently, participants underwent a comprehensive multidimensional evaluation, including the MASCoD screening, an extensive neuropsychological assessment, and neuroimaging (18F-FDG-PET).

### Participants

Participants were consecutively recruited from July 2022 until December 2024 from outpatient CDCDs for neurological evaluation of potential SCD at the *ICS Maugeri Montescano Institute* (Italy) (Neurophysiopathology Unit), the Neurology Unit of the *ASST Pavia - Voghera* (Italy). Inclusion criteria comprised self-reported SCD without diagnosed neurological or cognitive impairments, being adults aged 55 years or older, educational attainment in Italian, comprehension of research objectives, and voluntary non-remunerated participation. Exclusion criteria included severe medical conditions (e.g., significant cardiovascular, respiratory issues, neoplastic diseases), prior diagnosis of severe psychiatric disorders according to DSM-5 tr (American Psychiatric Association, 2022), diagnosed cognitive impairment, significant sensory deficits, or illiteracy.

The detection of SCD was based on anamnestic data collected by a neurologist with expertise in cognitive impairment during the clinical interview, utilizing questions that align with the Jessen criteria for SCD, for example by enquiring about the timing and circumstances of symptom onset (i.e whether cognitive decline emerged in the last 5 years or after the age of 60), exploring the presence of associated concerns or worries, assessing whether symptoms persist over time, establishing if medical assistance was sought, and determining whether an external observer has confirmed the existence of subjective cognitive decline (Jessen et al., 2014).

### Instruments

#### Multidimensional assessment of subjective cognitive decline

MASCoD is a brief screening battery developed through an expert consensus at the *ICS Maugeri Montescano Institute* (Italy) (Maffoni et al., 2022, MASCoD is included as Supplementary material).

MASCoD includes a first part collecting clinical and socio-demographic information, including family status, education, occupation, living situation, primary caregiver, BMI, smoking habits, and various anamnesis risk factors such as smoking, alcohol use, and addiction to drugs or psychostimulants. These data are already included in the routine clinical history interview, and this section of MASCoD enables the information to be recorded in a structured and organized manner.

Then, the MASCoD battery is constituted by three sections assessing:

(A) Comorbidities and risk factors thought 10 dichotomous answers (present/absence) according to Jessen criteria for SCD (Jessen et al., 2014).
(B) Subjective memory and attentional/executive difficulties in daily life. The 11 items require dichotomous answers (present/absence), but there is also a 4-point Likert scale for each item to qualitatively evaluate complaint intensity. To note, this section comprises items 1 to 6 from a slightly revised version of the widely used MAC-Q screening tool (Crook et al., 1992), adapted with the authors’ written consent. The modifications include updated, ecologically valid items better suited to the current generation—for example, recalling ‘passwords or other access codes’ instead of ‘telephone numbers.’ Additionally, this section has been expanded with new items targeting attentional and executive function difficulties.
(C) Anxiety or depressive symptoms through GAD-2 and PHQ-2 (Giuliani et al., 2021). This section, consisting of 4 items with 4-point Likert scale responses ranging from 0 (never) to 3 (almost every day), can be replaced by other screening tools for anxious and depressive symptoms. In addition, there is a single yes-no question assessing any positive (e.g., marriage, newborn) or negative (e.g., grief, job loss) distressing events occurring in the last year.

Overall, MASCoD can be considered a brief screening battery as it is constituted by different parts. Section A consists of a checklist covering well-established SCD risk factors and diagnostic criteria (Jessen et al., 2014), while Section C includes recommendations for using two previously validated brief screening tools (Giuliani et al., 2021). Section B represents the novel component, designed to explore symptoms and requiring psychometric validation.

Based on its theoretical foundation, the risk of developing cognitive impairment over time is determined by Section A, which assesses risk factors (yes = presence; no = absence), and Section B, which evaluates cognitive impairment (yes = presence of memory and attentional/executive difficulties; no = absence of these difficulties). Section C is utilized to aid in differential diagnosis and to identify potential co-occurring emotional disturbances.

However, the validation and specific cut-off of MASCoD are not yet available. Indeed, the assessment of psychometric characteristics is still in progress, and this work provides a preliminary contribution to the validation of this new screening tool.

Regarding administration, MASCoD was purposefully designed as a brief screening tool that can be easily administered by any healthcare professional without requiring specialized training. The development process involved multidisciplinary team meetings and item refinement precisely to ensure ease of use and practicality (Maffoni et al., 2022). Once fully validated, MASCoD could serve as a first-level screening instrument that general practitioners or other professionals can use to identify patients who require more in-depth evaluation and referral. Based on administrations conducted to date, the screening typically takes about 5–10 min, as some information is often already collected during the routine clinical anamnesis.

#### Neuropsychological tests

Participants underwent an extensive neuropsychological battery to both asses the clinical cognitive functioning following usual clinical practice suggested by DTCP and CCDDs standards (Frisoni et al., 2024), and to contribute to the process of validation of MASCoD:

- MMSE (Mini-Mental State Examination, Foderaro et al., 2022): widely used to assess global cognitive functioning. Italian normal cognitive functioning is ≥ 26.02 corrected score (maximum: 30).
- ACE-III (Addenbrooke’s Cognitive Examination III; Pigliautile et al., 2019): a comprehensive neuropsychological screening tool used to assess cognitive functions including attention, memory, verbal fluency, language, and visuospatial abilities. Italian normal cognitive functioning is ≥ 68.68 corrected score (maximum: 100).
- FAB (Frontal Assessment Battery, Aiello et al., 2022): evaluates executive functions including cognitive flexibility, planning, and inhibitory control. Italian normal functioning is ≥ 12.02 corrected score (maximum: 18).
- Semantic and Phonological Fluency (Costa et al., 2014): evaluates lexical access asking to the individual as much as possible words in 1 min per time on the bases of a semantic or phonological criterion. Italian clinical cut off for normal functioning is set on a corrected score ≥ 28.34 (Semantic Fluency) and ≥ 17.77 (Phonological fluency).
- TMT A and B (Trail Making Test A and B, Siciliano et al., 2019): assesses visual attention, processing psychomotor speed and visual scanning (Part A), and cognitive flexibility and task-switching (Part B). Italian normal functioning is ≤ 127 s (Part A) and ≤ 294 s (Part B).
- Stroop Test (Caffarra et al., 2002a): assesses selective attention, cognitive control, and inhibitory processes. Italian normal functioning is ≤ 36.91 s for time criterion and ≤ 4.23 for error criterion (maximum: 36).
- Digit Span Forward and Backward, and Corsi Block-Tapping Test (Forward and Backward): respectively measure verbal working memory and visuospatial working memory (Orsini et al., 1987). Italian clinical cut off for normal functioning is set on a corrected score: ≥ 4.26 (Digit Span Forward, maximum: 9), ≥ 2.65 (Digit Span Backword, maximum: 8), ≥ 3.46 (Corsi Forward, maximum: 9), ≥ 3.17 (Corsi Backword, maximum: 8).
- 15-word (Rey’s Auditory Verbal Learning Test, Carlesimo et al., 1996): measures verbal memory, specifically immediate recall and learning capacity (delayed recall). Italian clinical cut off for normal functioning is set on a corrected score ≥ 28.53 (15-word Immediate Recall, maximum: 75) and ≥ 4.69 (15-word Delayed Recall, maximum: 15).
- Rey Figure (Rey–Osterrieth Complex Figure - copy and recall, Caffarra et al., 2002b): assesses visuospatial construction and immediate and delayed memory. Italian clinical cut off for normal functioning is set on a corrected score ≥ 28.88 (maximum: 36).
- Raven Colored progressive Matrice CPM36 (>65 years, Measso et al., 1993) or Raven Progressive Matrice PM38 (< 65 years, Caffarra et al., 2003): evaluates non-verbal fluid intelligence and abstract reasoning capabilities. Italian normal functioning cut off is set on a corrected score ≥ 18.96 (Raven CPM36, maximum: 36) and ≥ 20.73 (Raven PM38, maximum: 48).
- CDT (Clock Drawing Test, Caffarra et al., 2011): evaluates visuospatial and executive functioning, often used for dementia screening. Italian normal functioning cut off is set on a corrected score ≥ 42.17 (maximum: 61).

#### Psychological assessment

Participants completed psychological screening tools:

- GAD-7 (Generalized Anxiety Disorder Scale, Spitzer et al., 2006; Kroenke et al., 2007): assesses anxiety symptoms, validated for Italian populations. Clinical cut off for mild anxious symptoms is set on a score of 5.
- PHQ-9 (Patient Health Questionnaire-9, Spitzer et al., 1999; Kroenke et al., 2001): evaluates depressive symptoms, with documented validity for Italian clinical and general populations. Clinical cut off for mild depressive symptoms is set on a score of 5.
- CFI (Cognitive Functional Index, Chipi et al., 2018): through 14 items measures functional cognition relevant to daily activities, applicable for identifying functional impairment due to cognitive deficits. The total score ranged between 0 and 12.5, with a mean of 3.30 ± 2.44.
  Although it is the gold standard and widely used, compared to MASCoD, the CFI focuses solely on everyday cognitive functioning and does not screen for anxiety or depressive symptoms, nor does it consider risk factors or other relevant comorbidities.

### Ethical consideration

The study received ethical approval from the *ICS Maugeri* Ethics Committee (Protocol CE 2666, 26 July 2022), in accordance with the principles of transparency, scientific rigor, and the Declaration of Helsinki. All participants provided written informed consent and were informed of their right to withdraw from the study at any time without providing a reason. No financial compensation was offered for participation.

This work was partially supported by the “Ricerca Corrente” funding scheme of the Ministry of Health, Italy.

### Statistical analysis

#### Aim 1

Descriptive and inferential analyses were conducted to characterize the sample and MASCoD scores.

Mean scores and standard deviations were reported for continuous variables, and frequency distributions for categorical variables.

We compared the group’s mean for each neuropsychological and psychological test score to a fixed benchmark (i.e., the normative cutoff) using one-sample t-tests.

Since no established clinical cutoff exists for the CFI, a provisional threshold was defined using a heuristic approach based on the mean and standard deviation reported in the original validation study (mean + 1 SD), as typically considered a possible primary sign of concern for any dysfunction in screening tool (Cascio and Barret, 2006).

#### Aim 2

Internal consistency reliability of MASCoD Section B was assessed using Kuder–Richardson Formula 20 (KR-20) to determine the influence of individual dichotomous items on the total scale reliability.

Pearson correlations were computed to assess convergent validity between the MASCoD and CFI, which is one of the most established and validated measure for SCD (Li et al., 2023) *(aim 2a)*.

Correlations were also calculated between MASCoD subscales and other cognitive and psychological measures (*aim 2b*).

#### Aim 3

To assess convergence between short and long forms in this population, paired sample t-tests compared the short (GAD-2, PHQ-2) and long (GAD-7, PHQ-9) forms of anxiety and depression assessments, examining differences in symptom severity estimation (*aim 3a*).

Finally, linear regression models, weighted by participants’ years of education, were employed to evaluate the predictive value of depressive symptoms (PHQ-9 scores) on SCD outcomes, including CFI, MASCoD (Section A, Section B, and the composite A + B scores) (*aim 3b*).

The Benjamini-Hochberg False Discovery Rate – FDR (Haynes, 2013) procedure was applied to control the expected proportion of false positives (type 1 error) by ranking *p*-values in ascending order, calculating critical values based on their rank and the total number of tests, and identifying the largest p-value meeting the threshold criterion (*q* = 0.05) to determine statistical significance.

All statistical analyses were conducted using Jamovi software, version 2.6. Significance was set at *p* < 0.05 (The Jamovi Project, 2025).

## Results

### Socio-demographic profile

The sample consisted of 59 participants who were predominantly older (69.36 ± 8.66), in retirement with moderate levels of education and a relatively high prevalence of cardiovascular risk factors (Table 1). Specifically, the mean age was 69.36 years (SD = 8.66), with an average of 10.05 years of education (SD = 3.83). Regarding marital status, the majority were married or cohabiting (61.0%), followed by widowed individuals (20.3%). Most participants were retired (64.4%), while smaller proportions were still full-time employees (11.9%). In terms of social support, nearly half of the participants (45.8%) reported their spouse/partner as the primary caregiver in case of need, followed by their children (37.3%). Concerning lifestyle habits, physical activity was reported by 33.9%, while an equal percentage reported no physical activity. Smoking behavior indicated that 13.6% were current smokers and 25.4% were former smokers.

**Table 1:** Descriptive characteristics of the sample.

Variables	Range	Mean	SD
Age	51–84	69.36	8.66
BMI	18.1–45.7	25.49	4.77
Years of education	5–19	10.05	3.83
	Counts		% of Total
Gender			
Female	42		71.2%
Male	17		28.8%
Marital Status			
Single	3		5.1%
Married/Cohabiting	36		61.0%
Widowed	12		20.3%
Separated/Divorced	8		13.6%
Employment Status			
Self-employed	4		6.8%
Full-time employee	7		11.9%
Part-time employee	4		6.8%
Homemaker	3		5.1%
Unemployed	3		5.1%
Retired	38		64.4%
Socio-family support			
Spouse/Partner	27		45.8%
Son/Daughter	22		37.3%
Other family member	2		3.4%
Nobody	8		13.6%
Lifestyle factors			
Physical activity			
Yes	20		33.9%
No	20		33.9%
Missing	19		32.2%
Smoking			
Yes	8		13.6%
No	32		54.2%
Former smoker	15		25.4%
Missing	4		6.8%
Alcohol			
Yes	7		11.9%
No	47		79.7%
Missing	5		8.5%
Psychoactive drug dependence*			
No	57		96.6%
Missing	2		3.4%
Clinical data			
Family history of cognitive disorders			
Yes	27		45.8%
No	20		33.9%
Missing	12		20.3%
Diabetes			
No	48		81.4%
Yes	9		15.3%
Missing	2		3.4%
Hyperuricemia			
No	57		96.6%
Missing	2		3.4%
Hypertension			
No	31		52.5%
Yes	26		44.1%
Missing	2		3.4%
Dyslipidemia			
No	47		79.7%
Yes	12		20.3%

*Psychoactive drugs refer here to anxiolytic medications or drugs with anticholinergic mechanisms, as well as substances of abuse. The prescription of benzodiazepines (including alprazolam and bromazepam) on an as-needed basis is not considered clinically relevant. Dependence is only taken into account if there is a current or past diagnosis of drug dependence or substance abuse disorder.

Regarding clinical and health conditions, the mean Body Mass Index (BMI) was 25.49 (SD = 4.77); nearly half (45.8%) of the participants reported a family history of cognitive disorders. Comorbidities included hypertension (45.6%), dyslipidemia (20.3%) and diabetes (15.8%), while none reported hyperuricemia. No participants reported psychoactive substance or drug dependence.

### Neuropsychological and psychological performance

The neuropsychological profile of the sample revealed cognitive performance significantly above the population minimum normative values across all domains (Table 2), as expected in SCD. Indeed, the fixed benchmark values used for comparison represent the lower limit of normal performance, corresponding to the clinical cut-off for normal functioning, that is equivalent score = 1 (Facchin et al., 2022).

**Table 2:** Neuropsychological and psychological data from our sample and normative data.

Test	Normative value	Mean	SD	One-sample *t* test	*p*-value
Neuropsychological tests					
MMSE	≥ 26.02	29.0	1.24	−18.46	<0.001
ACE-III	≥ 68.68	86.47	7.33	−17.33	<0.001
FAB	≥ 12.02	15.3	2.14	−11.77	<0.001
Phonological fluency	≥ 17.77	35.98	9.18	15.23	<0.001
Semantic fluency	≥ 28.34	46.29	9.31	14.81	<0.001
TMT A	≤ 127.0	36.68	20.08	−34.55	<0.001
TMT B	≤ 294.0	99.82	54.19	−27.05	<0.001
Stroop time	≤ 36.91	18.81	10.85	−12.59	<0.001
Stroop errors	≤ 4.23	1.39	3.74	−5.77	<0.001
Digit span forward	≥ 4.26	5.8	1.01	11.77	<0.001
Digit span backward	≥ 2.65	4.29	1.13	10.28	<0.001
Corsi forward	≥ 3.46	5.24	1.32	10.36	<0.001
Corsi backward	≥ 3.17	4.6	1.46	7.54	<0.001
15-word immediate recall	≥ 28.53	42.3	11.0	9.62	<0.001
15-word delayed recall	≥ 4.69	9.12	3.98	8.55	<0.001
Rey figure recall	≥ 9.47	16.22	6.14	8.44	<0.001
Rey figure copy	≥ 28.88	32.15	5.81	4.32	<0.001
Raven CPM36	≥ 18.96	30.39	3.82	18.69	<0.001
Raven PM38	≥ 20.73	35.51	7.17	9.22	<0.001
CDT	≥ 42.17	57.25	6.33	18.28	<0.001
Psychological tests					
CFI	≥ 5.74*	5.10	2.52	−2.03	0.048
PHQ-9	≥ 5.0	6.76	5.16	2.62	0.011
GAD-7	≥ 5.0	5.76	4.31	1.36	0.179

MASCoD					
MASCoD	Min-Max	IQR	Mean	SD	Score range
Section A	3–8	2	5.93	1.33	0–10
Section B	0–11	3	5.47	2.86	0–11
Section A + B	4–17	4	11.42	3.43	0–21
Section C_GAD2	0–6	2	2.23	1.74	0–6
Section C_PHQ2	0–6	2	1.70	1.46	0–6

*A preliminary clinical cutoff was set at mean + 1 standard deviation (3.30 + 2.44 = 5.74), based on the clinical assumption that scores above the average may indicate potential cognitive impairment, and in line with a heuristic approach (Cascio and Barret, 2006). Min-Max, minimum scores-maximum score; IQR, interquartile range; SD, standard deviation.

Concerning the psychological self-report measures (Table 2), the 38.98% of the sample exceeded the clinical threshold (mean + 1 standard deviation) on CFI, indicating the presence of SCD. Overall, the sample reported slightly greater subjective functional complaints (CFI) compared to the general population, with the difference reaching marginal statistical significance (sample mean = 5.10, *p* = 0.05). Considering the mild severity threshold for depressive symptoms (PHQ-9 score = 5), the sample reported greater symptomatology compared to the general population (sample mean = 6.76, *p* = 0.01). In contrast, anxiety levels were within normal limits and did not significantly differ from population values (GAD-7 mild severity threshold = 5, sample mean = 5.76, *p* = 0.18).

Concerning descriptive analysis of the MASCoD (Table 2), Section A (risk factors) had a mean score of 5.93 ± 1.33 out of a maximum of 10, while Section B (memory and attention subjective complaints) averaged 5.47 ± 2.86 out of 11. The combined score of Sections A and B yielded a mean of 11.42 ± 3.43 out of 21. Section C, addressing psychological symptoms, revealed a mean score of 2.23 ± 1.74 on the GAD-2 and 1.70 ± 1.46 on the PHQ-2.

### Internal consistency reliability of MASCoD

As Section B of MASCoD is the novel component requiring validation, we assess the internal consistency of this section through Kuder–Richardson Formula 20 (KR-20) (Table 3).

**Table 3:** Internal consistency of MASCoD Section B.

Scale reliability statistic	K-20 index
Overall scale	0.782
Item reliability statistics	K-20 index if item dropped
MASCoD_section B item1	0.747
MASCoD_section B item 2	0.755
MASCoD_ section B item 3	0.766
MASCoD_ section B item 4	0.761
MASCoD_ section B item 5	0.754
MASCoD_ section B item 6	0.765
MASCoD_ section B item 7	0.785
MASCoD_ section B item 8	0.777
MASCoD_ section B item 9	0.759
MASCoD_ section B item 10	0.781
MASCoD_ section B item 11	0.766

The overall reliability coefficient obtained was KR-20 = 0.782, indicating good internal consistency supporting its reliability for subsequent analysis. Furthermore, item-level reliability analysis revealed that the removal of individual items had minimal impact on the overall reliability coefficient, ranging from 0.747 to 0.785.

### Convergent validity of MASCoD

Table 4 presents the Pearson correlation coefficients among the different sections of the MASCoD and the CFI self-report.

**Table 4:** Pearson correlations among the different sections of the MASCoD and the CFI.

Correlations	MASCoD Section A	MASCoD Section B	MASCoD Section A + B	MASCoD Section C (GAD2)	MASCoD Section C (PHQ2)	CFI self-report
MASCoD Section A	–					
MASCoD Section B	0.224	–				
MASCoD Section A + B	0.580***	0.923***	–			
MASCoD Section C (GAD2)	0.260	−0.036	0.070	–		
MASCoD Section C (PHQ2)	0.108	0.047	0.082	0.531***	–	
CFI self-report	0.274	0.435**	0.473***	0.021	0.072	–

*p < 0.05; **p < 0.01; ***p < 0.001.

Strong positive correlations were observed between MASCoD Section A and the combined Section A + B (*r* = 0.580, *p* < 0.001), and between MASCoD Section B and Section A + B (*r* = 0.923, *p* < 0.001). Moreover, a positive correlation also emerges between GAD-2 and PHQ-9 (*r* = 0.531, *p* < 0.001).

The analysis revealed a moderate and statistically significant correlation between the CFI self-report and MASCoD Section B (*r* = 0.435, *p* = 0.01), as well as the combined Section A + B (*r* = 0.473, *p* < 0.001). The correlation between CFI and Section A was weaker and only approached significance (*r* = 0.274, *p* = 0.063). Correlations between CFI and other MASCoD Section C were non-significant, including Section C GAD-2 (*r* = 0.021, *p* = 0.887) and Section C PHQ-2 (*r* = 0.072, *p* = 0.631).

### Correlations analysis between variables for construct validity of MASCoD

The correlational analysis between MASCoD and CFI self-report versus a battery of neuropsychological and psychopathological measures yielded some statistically significant findings (See Appendix A, B).

#### Multidimensional assessment of subjective cognitive decline

A significant negative correlation between MASCoD Section B and ACE-III performance (*r* = −0.342, *p* = 0.015) indicates that greater SCD is associated with poorer global cognitive performance.

Conversely, Section A of the MASCoD was negatively correlated with TMT-A performance (*r* = −0.320, *p* = 0.015).

Both MASCoD Section A (*r* = 0.327, *p* = 0.013) and the composite MASCoD score A + B (*r* = 0.299, *p* = 0.024) were positively associated with depressive symptoms as measured by the PHQ-9.

#### CFI self-report

First, a moderate and negative correlation was observed between the CFI self-report and the Corsi Backward test (*r* = −0.293, *p* = 0.041), suggesting that greater perceived SCD—as captured by the CFI—is associated with lower performance on backward visuospatial working memory.

Second, a positive and statistically significant correlation emerged between the CFI self-report and PHQ-9 depressive symptomatology (*r* = 0.328, *p* = 0.022), indicating that individuals reporting higher levels of SCD also tend to endorse more clinically relevant depressive symptoms.

### Convergence between short and long version of GAD and PHQ

Statistical comparison with longer versions of the same scales showed that GAD-7 scores were significantly higher than GAD-2 scores (*t* = 5.54, *p* < 0.001), and the same for PHQ-9 scores versus PHQ-2 scores (*t* = 8.54, *p* < 0.001; Table 5).

**Table 5:** Comparison of GAD and PHQ scores.

Comparison	Mean long version	Mean short version	Δ mean	*t*-value	*p*-value
GAD	5.76	2.23	3.53	6.87	<0.001
PHQ	6.76	1.70	5.06	8.21	<0.001

### Predictivity of depressive symptoms to SCD

Given that depressive symptoms were the only variable significantly associated with all measures of subjective cognitive decline (SCD), we conducted linear regression analyses to examine whether depressive symptoms could predict levels of SCD (Table 6). Since years of education varied widely among participants (ranging from 5 to 19), the regression was weighted by education to account for differences in educational attainment.

**Table 6:** Regressions models.

Dependent variable	*R*	*R* ^2^	PHQ-9 Estimate (β)	SE	*t*	*p*	(FDR 0.05)	
							Rank	Critical value
CFI self-report	0.314	0.099	0.159	0.070	2.27	0.028	3	0.0375
MASCoD Section A	0.354	0.125	0.091	0.032	2.80	0.007	1	0.0125
MASCoD Section B	0.254	0.065	0.126	0.065	1.95	0.057	4	0.05
MASCoD Total (A + B)	0.348	0.121	0.212	0.077	2.76	0.008	2	0.025

Considering MASCoD as outcome, a first model was significant (*R*^2^ = 0.107, *p* = 0.013): PHQ-9 scores positively predicted higher SCD assessed with MASCoD Section A (*β* = 0.0834, *p* = 0.013).

The combined MASCoD score A + B was also significantly predicted by depressive symptoms. The model accounted for nearly 9% of the variance (*R*^2^ = 0.0896, *p* = 0.024), with PHQ-9 scores emerging as a significant predictor (*β* = 0.196, *p* = 0.024).

Although a positive association was observed between PHQ-9 scores and MASCoD Section B (*β* = 0.117), this did not reach statistical significance (*p* = 0.112).

A similar pattern emerged in the regression model with CFI as the outcome. The regression model predicting CFI scores from PHQ-9 was statistically significant (*R*^2^ = 0.107, *p* = 0.022), explaining approximately 11% of the variance in SCD. That is, higher PHQ-9 scores significantly predicted greater SCD as measured by the CFI (*β* = 0.166, *p* = 0.022), indicating that increased depressive symptoms are associated with more pronounced self-reported cognitive decline.

Statistical significance of these regression models was adjusted for multiple comparisons using the Benjamini-Hochberg False Discovery Rate (FDR) method at *q* = 0.05 (5%) (Haynes, 2013). Applying this correction, MASCoD Section A, MASCoD Total, and CFI self-report remain significant, while MASCoD Section B is set to 0.05.

## Discussion

The present study was conducted in response to growing evidence emphasizing the heterogeneity of SCD, still creating disparities among studies (Molinuevo et al., 2017), as well as the pressing need for shared reliable, multidimensional tools to detect and monitor it (Rabin et al., 2015).

Firstly, our study provides important descriptive insights into the sociodemographic characteristics of a SCD sample consisting predominantly of older if compared to earlier studies (Jessen et al., 2014; Rabin et al., 2017), retired individuals with moderate educational levels and notable cardiovascular risk factors. Given the limited sample size of 59 participants, caution is warranted when drawing comparisons with much larger cohorts, such as the recent U. S.-based Brain Health Registry (BHR) cohort (*N* = 27,596) (Tank et al., 2024). Nonetheless, some general similarities in age, gender distribution, and family history of cognitive disorders are observed. Conversely, our Italian sample had lower educational levels, possibly due to cultural or generational differences, and showed limited physical activity, low alcohol and tobacco use, strong family support, and higher rates of hypertension, diabetes, and dyslipidemia—indicating greater vascular risk burden compared to the BHR cohort (Tank et al., 2024). Other studies indicate that cardiovascular health significantly influences cognitive outcomes in older adults (Gorelick et al., 2011; Qiu and Fratiglioni, 2015). Specifically, hypertension, observed in nearly half of our participants, is a well-established modifiable risk factor for cognitive decline, thus emphasizing the importance of early monitoring and intervention in this population (Iadecola et al., 2016). Overall, these differences suggest that the cohorts may represent individuals shaped by distinct underlying factors, further potentially influenced by cultural context and cohort-specific historical experiences (Jessen et al., 2014; Zhang et al., 2021; Arora et al., 2024). Thus, these characteristics highlight the relevance of socioeconomic and healthcare contexts in shaping the expression and potential risk pathways of SCD. Further research should explore cognitive reserve and lifestyle factors potentially mitigating the progression to objective cognitive impairment in this older subgroup (Arora et al., 2024).

Regarding cognitive and psychological performance, neuropsychological assessments showed cognitive performance consistently higher-than-normative benchmarks across various domains, reinforcing the notion that subjective cognitive complaints frequently do not correlate directly with objective cognitive deficits (Zlatar et al., 2014; Buckley et al., 2015; Hill et al., 2016; Zlatar et al., 2018; Hill et al., 2021). A key challenge in clinical and research settings for SCD is indeed the lack of standardized diagnostic criteria, especially neuropsychological test thresholds. Diagnosis rates vary widely depending on the criteria and tests used, with stricter thresholds identifying individuals with greater impairment (Pestana et al., 2024), and efforts continue to balance the use of more demanding cognitive tasks (De Simone et al., 2023) with more restrictive assessment tools to enhance diagnostic reliability (Rabin et al., 2015).

On the one hand, although cognitive performance was expected to be within the normal range, this finding prompts a deeper reflection on the adequacy of current neuropsychological assessment tools in capturing the subtle cognitive changes reported by individuals with SCD. This observation suggests that standard cognitive tests, although widely used in clinical practice, may not be sufficiently sensitive to detect subclinical or early-stage cognitive deficits, indicating the potential value of integrating more sensitive, functional, or ecologically valid measures that can capture early subjective cognitive changes, which may still be noticeable to individuals familiar with their baseline functioning (González et al., 2021). On the other hand, the high performance across executive functions, memory, and visuospatial tasks underscores that SCD in our sample is likely reflective of heightened self-awareness and metacognition (Zhang et al., 2021; Cappa et al., 2024) or psychological symptoms rather than genuine cognitive dysfunction. In this regard, the presence of mild depressive symptoms identified in our sample predicts the subjective cognitive complaints, explaining around 9–11% of variance, both considering MASCoD (Section A and A + B) or CFI, so highlighting the critical role of psychological health in perceived cognitive decline. This result emphasizes the necessity of addressing depressive symptoms in clinical evaluations and interventions for older adults with SCD. These findings are consistent with previous studies suggesting that emotional factors significantly influence subjective cognitive assessments (Zlatar et al., 2014; Burmester et al., 2016; Hill et al., 2016; Molinuevo et al., 2017; Zlatar et al., 2018; Hill et al., 2021). Specifically, Zlatar et al. (2018) found no significant link between SCD and objective cognition after adjusting for depression, while SCD consistently correlated with depression symptoms. Hill et al. (2021) reported that depressive symptoms partially mediated the relationship between SCD and objective memory in three out of four datasets. A review by the same author strengthens the possibility that SCD may reflect subtle depressive symptoms rather than objective cognitive dysfunction or anxious symptoms (Hill et al., 2016). Although preliminary, these data reinforce the link of SCD more with psychological aspects rather than cognitive functioning, consistent with previous literature highlighting the complexity and multifaceted nature of SCD (Hill et al., 2021; Zlatar et al., 2014; Hill et al., 2016; Molinuevo et al., 2017; Zlatar et al., 2018).

The literature remains inconclusive regarding the most appropriate screening tools for assessing depressive and anxiety symptoms, particularly across diverse clinical populations. Although brief screening instruments such as the PHQ-2 and GAD-2 have demonstrated acceptable reliability and validity (Kroenke et al., 2003, 2009; Arroll et al., 2010; Plummer et al., 2016; Staples et al., 2019; Giuliani et al., 2021), their psychometric performance relative to their full-length counterparts—the PHQ-9 and GAD-7—has not been comprehensively evaluated across all settings (Lang et al., 2009). These abbreviated tools offer clear practical advantages in time-constrained clinical environments, notably improving response rates and allowing integration into broader screening protocols. However, this same feature may limit their sensitivity in populations with subtler or less overt symptomatology, such as individuals with SCD or chronic pain (Bisby et al., 2022). Consistent with this hypothesis, our findings revealed notable discrepancies between the short forms (PHQ-2 and GAD-2) and the full-length measures (PHQ-9 and GAD-7) within the SCD sample. These results suggest that while brief instruments may serve as useful preliminary screening tools, they risk underestimating the severity of affective symptoms in this population. Accordingly, comprehensive psychological assessments should complement brief cognitive screenings to ensure accurate detection of emotional symptoms in older adults with SCD.

Concerning the new screening battery called MASCoD, it is important to first discuss the similarities and differences with the gold standard CFI. Both instruments target the earliest, subjective stage of cognitive decline, yet each spotlights different facets. Specifically, MASCoD is organized into three sections that collect separate streams of information: SCD-plus risk features according to definition of the condition (Jessen et al., 2014), (Section A); everyday memory, attentional and executive complaints (Section B); and ultra-brief mood screens (GAD-2, PHQ-2, Giuliani et al., 2021) plus a yes/no question about stressful life events in the past year (Section C). All items are dichotomous for ease of use, while Section B adds a 4-point Likert scale to indicate the severity of each complaint. After full psychometric validation, MASCoD will stratify patients into low, medium or high risk and recommend specific follow-up intervals or neuropsychological referral. By contrast, the CFI is a 14-item self-report questionnaire scored Yes/Maybe/No that tracks year-to-year functional change, omitting mood assessment and lacking different cut-off thresholds. Thus, CFI is more commonly used and is useful for longitudinal monitoring of perceived functional decline, whereas MASCoD may be better suited for initial clinical triage because it integrates affective context and provides explicit management guidance.

This preliminary contribution to MASCoD validation demonstrated satisfactory internal consistency reliability and good correlations with CFI, supporting its use as a reliable screening instrument. Specifically, MASCoD Section B and the combined A + B measure demonstrate the strongest convergent validity with the CFI self-report, while the anxiety and depression components (Section C) appear to measure distinct constructs with limited overlap with the CFI measure. It could be explained by the fact that CFI assesses more cognitive and behavioral nuances of subjective functioning, neglecting the emotional sphere.

Moreover, higher scores on MASCoD Section B were significantly associated with lower ACE-III performance, indicating reduced global cognitive functioning, although not yet in the pathological range. Interestingly, a significant negative correlation was also observed between MASCoD Section A scores and TMT-A performance. Since lower TMT-A scores reflect better attention and processing speed, this result suggests that individuals reporting greater SCD may demonstrate enhanced attentional control or compensatory cognitive strategies thanks to the fact that executive and attentional functions are preserved and this, in turn, might mask underlying difficulties (Burmester et al., 2016). These mechanisms may reflect heightened patient’s cognitive vigilance not only toward potential risk factors and the need for medical consultation (MASCoD Section A), but also during cognitively demanding tasks, driven by increased self-awareness (Burmester et al., 2016; Zhang et al., 2021; Cappa et al., 2024). To this regard, it has to notice that our SCD cohort can be conceptualized as a self-referred, autonomous sample, as participants voluntarily sought evaluation in the absence of external recruitment strategies, a pattern already well-documented in memory-clinic (Jessen et al., 2020; Zhang et al., 2021). This phenomenon likely reflects shifting sociocultural dynamics: there is increasing emphasis on personal health monitoring, not only in terms of physical well-being—such as widespread cancer screening—but also with growing attention to cognitive and mental health. The traditional narrative of inevitable cognitive impairment in older age is gradually being replaced by a more nuanced understanding that recognizes the potential for healthy aging. Cognitive decline is no longer viewed merely as a taboo topic; although certain beliefs and socio-cultural barriers persist, it is increasingly recognized as a legitimate area for proactive engagement, promoting a more health-literate and prevention-oriented aging population (Zhang et al., 2021; Allen and Sikora, 2023).

Overall, the observed pattern of associations between cognitive measures and MASCoD dimensions likely reflects the multifaceted nature of the subjective experience of cognitive decline. This experience may differ depending on underlying etiologies—for instance, whether the SCD profile is primarily associated with emotional factors or represents an early manifestation of preclinical Alzheimer’s disease (Jessen et al., 2014, 2020). In some individuals, preserved insight and heightened metacognitive abilities may enable the detection of subtle cognitive changes relative to their personal baseline, even in the absence of objective impairment (Rabin et al., 2015; Cappa et al., 2024).

## Limits, strengths and future directions

This study contributes to the comprehensive characterization of the SCD population by collecting detailed data on health, lifestyle, and demographic variables, and by employing robust, validated neuropsychological and psychological assessment tools. In response to the urgent need for harmonization of criteria and instruments in the management of SCD, it also offers a preliminary contribution to the validation of MASCoD—a novel, brief, and multidimensional screening tool—which demonstrated good internal consistency and convergent validity. Nonetheless, the study is subject to certain limitations, including a small sample size that restricts generalizability, a cross-sectional design that limits causal inference, and potential biases inherent to self-report measures or characteristics of chosen tests. Furthermore, a more structured psychometric evaluation involving larger samples is required to confirm the clinical utility of MASCoD. Another consideration is that a significant proportion of the variance in MASCoD scores was explained by depressive symptoms, indicating that the tool may capture psychological distress alongside early cognitive decline. This overlap may reduce the specificity of MASCoD in distinguishing between affective symptoms and true neurocognitive impairment. Therefore, results should be interpreted within a comprehensive clinical context, and further longitudinal studies are needed to clarify MASCoD’s discriminative validity and improve its specificity in detecting early cognitive changes.

Cultural, personality traits (here not assessed) and educational specificity of the sample may also limit the broader applicability of the findings, underscoring the need for further cross-cultural validation.

Finally, future research should not only address the limits mentioned above but also investigate the roles of cognitive reserve and lifestyle factors. Although our sample exhibits higher cardiovascular risk factors, it also appears to benefit from certain potentially protective lifestyle characteristics, including greater social support, moderate educational attainment, and low levels of alcohol and tobacco use. These factors may contribute to building cognitive reserve, possibly enabling individuals to maintain a heightened awareness of their own cognitive functioning throughout life and, consequently, to be more sensitive in detecting subtle or subclinical changes in their cognitive performance. While this remains a hypothesis that requires further investigation, it underscores the importance of future research focusing on lifestyle factors such as diet, social relationships, and physical activity, as well as social support, as potential preventive measures and intervention targets against cognitive decline. Indeed, existing literature suggests that such elements play a critical role in enhancing cognitive reserve and mitigating the impact of neurodegenerative processes (Röhr et al., 2020; Arora et al., 2024).

## Conclusion

This study contributes to the growing body of research on SCD by offering a multidimensional characterization of this population. Despite the presence of subjective cognitive complaints, participants demonstrated preserved cognitive performance, reinforcing the dissociation between perceived and objective impairment. Depressive symptoms emerged as significant predictors of SCD, underscoring the need for integrated affective screening in SCD assessment. Our findings also highlight the limitations of ultra-brief screening tools in detecting subtle emotional symptoms in this population. Finally, preliminary contribution to the validation of the MASCoD battery supports its reliability and convergent validity with established SCD measures. Overall the complex interplay between self-awareness, emotional health, and cognitive function in SCD calls for longitudinal research to clarify underlying mechanisms and to inform early, targeted interventions, as for example rehabilitation ones.

Overall, these findings underscore the complexity of interpreting SCD, suggesting the need for comprehensive cognitive, psychological, and medical evaluations to better differentiate SCD profiles, which is essential for proposing tailored preventive, supportive, and potentially rehabilitation interventions.

## Data Availability

The raw data supporting the conclusions of this article will be made available by the authors, without undue reservation.

## References

[ref1] Aiello E. N. Esposito A. Gramegna C. Gazzaniga V. Zago S. Difonzo T.. (2022). The frontal assessment battery (FAB) and its sub-scales: validation and updated normative data in an Italian population sample. Neurol. Sci. 43, 979–984. doi: 10.1007/s10072-021-05392-y, PMID: 34184168 PMC8789707

[ref2] Allen J. O. Sikora N. (2023). Aging stigma and the health of US adults over 65: what do we know? Clin. Interv. Aging 18, 2093–2116. doi: 10.2147/CIA.S396833, PMID: 38116457 PMC10729833

[ref3] Amariglio R. (2021). Putting memory complaints in context matters. Int. Psychogeriatr. 33, 645–646. doi: 10.1017/S1041610220001672, PMID: 34308816

[ref4] American Psychiatric Association (2022). Manuale diagnostico e statistico dei disturbi mentali – Quinta edizione, edizione rivista (DSM-5-TR).Tr.it. Milano: Raffaello Cortina Editore.

[ref5] Arora S. Patten S. B. Mallo S. C. Lojo-Seoane C. Felpete A. Facal-Mayo D.. (2024). The influence of education in predicting conversion from subjective cognitive decline (SCD) to objective cognitive impairment: a systematic review and meta-analysis. Ageing Res. Rev. 101:102487. doi: 10.1016/j.arr.2024.102487, PMID: 39243892

[ref6] Arroll B. Goodyear-Smith F. Crengle S. Gunn J. Kerse N. Fishman T.. (2010). Validation of PHQ-2 and PHQ-9 to screen for major depression in the primary care population. Ann. Fam. Med. 8, 348–353. doi: 10.1370/afm.1139, PMID: 20644190 PMC2906530

[ref7] Bacigalupo I. Giaquinto F. Salvi E. Carnevale G. Vaccaro R. Matascioli F.. (2024). A new national survey of centers for cognitive disorders and dementias in Italy. Neurol. Sci. 45, 525–538. doi: 10.1007/s10072-023-06958-8, PMID: 37592124 PMC10791890

[ref8] Bisby M. A. Karin E. Scott A. J. Dudeney J. Fisher A. Gandy M.. (2022). Examining the psychometric properties of brief screening measures of depression and anxiety in chronic pain: the patient health questionnaire 2-item and generalized anxiety disorder 2-item. Pain Pract. 22, 478–486. doi: 10.1111/papr.13107, PMID: 35258171 PMC9311649

[ref9] Buckley R. F. Ellis K. A. Ames D. Rowe C. C. Lautenschlager N. T. Maruff P.. (2015). Phenomenological characterization of memory complaints in preclinical and prodromal Alzheimer’s disease. Neuropsychology 29, 571–581. doi: 10.1037/neu0000156 25664464

[ref10] Burmester B. Leathem J. Merrick P. (2016). Subjective cognitive complaints and objective cognitive function in aging: a systematic review and Meta-analysis of recent cross-sectional findings. Neuropsychol. Rev. 26, 376–393. doi: 10.1007/s11065-016-9332-2, PMID: 27714573

[ref11] Caffarra P. Gardini S. Zonato F. Concari L. Dieci F. Copelli S.. (2011). Italian norms for the Freedman version of the clock drawing test. J. Clin. Exp. Neuropsychol. 33, 982–988. doi: 10.1080/13803395.2011.589373, PMID: 22082081

[ref12] Caffarra P. Vezzadini G. Dieci F. Zonato F. Venneri A. (2002a). Rey-Osterrieth complex figure: normative values in an Italian population sample. Neurol. Sci. 22, 443–447. doi: 10.1007/s100720200003, PMID: 11976975

[ref13] Caffarra P. Vezzadini G. Francesca D. Zonato F. Venneri A. (2002b). A short version of the Stroop test: normative data in an Italian population sample. Nuova Riv. Neurol. 12, 111–115.

[ref14] Caffarra P. Vezzadini G. Zonato F. Copelli S. Venneri A. (2003). A normative study of a shorter version of raven's progressive matrices 1938. Neurol. Sci. 24, 336–339. doi: 10.1007/s10072-003-0185-0, PMID: 14716529

[ref15] Cappa S. F. Ribaldi F. Chicherio C. Frisoni G. B. (2024). Subjective cognitive decline: memory complaints, cognitive awareness, and metacognition. Alzheimers Dement. 20, 6622–6631. doi: 10.1002/alz.13905, PMID: 39051174 PMC11497716

[ref16] Carlesimo G. A. Caltagirone C. Gainotti G. (1996). The mental deterioration battery: normative data, diagnostic reliability and qualitative analyses of cognitive impairment The Group for the Standardization of the Mental Deterioration Battery. Eur. Neurol. 36, 378–384. doi: 10.1159/000117297, PMID: 8954307

[ref17] Cascio W. F. Barret G. (2006). Setting cutoff scores: legal, psychometric, and professional issues and guidelines. Pers. Psychol. 41, 1–24. doi: 10.1111/j.1744-6570.1988.tb00629.x

[ref18] Chipi E. Frattini G. Eusebi P. Mollica A. D'Andrea K. Russo M.. (2018). The Italian version of cognitive function instrument (CFI): reliability and validity in a cohort of healthy elderly. Neurol. Sci. 39, 111–118. doi: 10.1007/s10072-017-3150-z, PMID: 29063452

[ref19] Costa A. Bagoj E. Monaco M. Zabberoni S. De Rosa S. Papantonio A. M.. (2014). Standardization and normative data obtained in the Italian population for a new verbal fluency instrument, the phonemic/semantic alternate fluency test. Neurol. Sci. 35, 365–372. doi: 10.1007/s10072-013-1520-8, PMID: 23963806

[ref20] Crook T. H. Feher E. P. Larrabee G. J. (1992). Assessment of memory complaint in age-associated memory impairment: the MAC-Q. Int. Psychogeriatr. 4, 165–176. doi: 10.1017/s1041610292000991, PMID: 1477304

[ref21] De Simone M. S. Rodini M. De Tollis M. Fadda L. Caltagirone C. Carlesimo G. A. (2023). The diagnostic usefulness of experimental memory tasks for detecting subjective cognitive decline: preliminary results in an Italian sample. Neuropsychology 37, 636–649. doi: 10.1037/neu0000846, PMID: 35980693

[ref22] Facchin A. Rizzi E. Vezzoli M. (2022). A rank subdivision of equivalent score for enhancing neuropsychological test norms. Neurol. Sci. 43, 5243–5249. doi: 10.1007/s10072-022-06140-6, PMID: 35581425 PMC9385822

[ref23] Foderaro G. Isella V. Mazzone A. Biglia E. Di Gangi M. Pasotti F.. (2022). Brand new norms for a good old test: northern Italy normative study of MiniMental state examination. Neurol. Sci. 43, 3053–3063. doi: 10.1007/s10072-021-05845-4, PMID: 34989910 PMC9018649

[ref24] Frisoni G. B. Festari C. Massa F. Cotta Ramusino M. Orini S. Aarsland D.. (2024). European intersocietal recommendations for the biomarker-based diagnosis of neurocognitive disorders. Lancet Neurol. 23, 302–312. doi: 10.1016/S1474-4422(23)00447-7, PMID: 38365381

[ref25] Giuliani M. Gorini A. Barbieri S. Veglia F. Tremoli E. (2021). Examination of the best cut-off points of PHQ-2 and GAD-2 for detecting depression and anxiety in Italian cardiovascular inpatients. Psychol. Health 36, 1088–1101. doi: 10.1080/08870446.2020.1830093, PMID: 33026888

[ref26] González D. A. Gonzales M. M. Jennette K. J. Soble J. R. Fongang B. (2021). Cognitive screening with functional assessment improves diagnostic accuracy and attenuates bias. Alzheimers Dement. 13:e12250. doi: 10.1002/dad2.12250, PMID: 34934799 PMC8652409

[ref27] Gorelick P. B. Scuteri A. Black S. E. Decarli C. Greenberg S. M. Iadecola C.. (2011). Vascular contributions to cognitive impairment and dementia: a statement for healthcare professionals from the American Heart Association/American Stroke Association. Stroke 42, 2672–2713. doi: 10.1161/STR.0b013e3182299496, PMID: 21778438 PMC3778669

[ref28] Haynes W. (2013). “Benjamini–Hochberg Method” in Encyclopedia of systems biology. eds. Dubitzky W. Wolkenhauer O. Cho K. H. Yokota H. (New York: Springer).

[ref29] Hill N. L. Bhargava S. Bratlee-Whitaker E. Turner J. R. Brown M. J. Mogle J. (2021). Longitudinal relationships between subjective cognitive decline and objective memory: depressive symptoms mediate between-person associations. J. Alzheimers Dis. 83, 1623–1636. doi: 10.3233/JAD-210230, PMID: 34420951 PMC8787869

[ref30] Hill N. L. Mogle J. Wion R. Munoz E. DePasquale N. Yevchak A. M.. (2016). Subjective cognitive impairment and affective symptoms: a systematic review. The Gerontologist 56, e109–e127. doi: 10.1093/geront/gnw091, PMID: 27342440 PMC5181393

[ref31] Holmen J. Langballe E. Midthjell K. Lingaas H. T. Fiksaune A. Saltvedt I.. (2013). Gender differences in subjective memory impairment in a general population: the HUNT study, Norway. BMC Psychol. 1:19. doi: 10.1186/2050-7283-1-19

[ref32] Iadecola C. Yaffe K. Biller J. Bratzke L. C. Faraci F. M. Gorelick P. B.. (2016). Impact of hypertension on cognitive function: a scientific statement from the American Heart Association. Hypertension (Dallas, Tex.: 1979) 68:e67. doi: 10.1161/HYP.0000000000000053, PMID: 27977393 PMC5361411

[ref33] Ibnidris A. Robinson J. N. Stubbs M. Piumatti G. Govia I. Albanese E. (2022). Evaluating measurement properties of subjective cognitive decline self-reported outcome measures: a systematic review. Syst. Rev. 11:144. doi: 10.1186/s13643-022-02018-y, PMID: 35850915 PMC9290248

[ref34] Jessen F. Amariglio R. E. Buckley R. F. van der Flier W. M. Han Y. Molinuevo J. L.. (2020). The characterisation of subjective cognitive decline. Lancet Neurol. 19, 271–278. doi: 10.1016/S1474-4422(19)30368-0, PMID: 31958406 PMC7062546

[ref35] Jessen F. Amariglio R. E. van Boxtel M. Breteler M. Ceccaldi M. Chételat G.. (2014). A conceptual framework for research on subjective cognitive decline in preclinical Alzheimer's disease. Alzheimers Dement. 10, 844–852. doi: 10.1016/j.jalz.2014.01.001, PMID: 24798886 PMC4317324

[ref36] Kroenke K. Spitzer R. L. Williams J. B. (2001). The PHQ-9: validity of a brief depression severity measure. J. Gen. Intern. Med. 16, 606–613. doi: 10.1046/j.1525-1497.2001.016009606.x, PMID: 11556941 PMC1495268

[ref37] Kroenke K. Spitzer R. L. Williams J. B. (2003). The patient health questionnaire-2: validity of a two-item depression screener. Med. Care 41, 1284–1292. doi: 10.1097/01.MLR.0000093487.78664.3C, PMID: 14583691

[ref38] Kroenke K. Spitzer R. L. Williams J. B. Löwe B. (2009). An ultra-brief screening scale for anxiety and depression: the PHQ-4. Psychosomatics 50, 613–621. doi: 10.1176/appi.psy.50.6.613, PMID: 19996233

[ref39] Kroenke K. Spitzer R. L. Williams J. B. Monahan P. O. Löwe B. (2007). Anxiety disorders in primary care: prevalence, impairment, comorbidity, and detection. Ann. Intern. Med. 146, 317–325. doi: 10.7326/0003-4819-146-5-200703060-00004, PMID: 17339617

[ref40] Lang A. J. Norman S. B. Means-Christensen A. Stein M. B. (2009). Abbreviated brief symptom inventory for use as an anxiety and depression screening instrument in primary care. Depress. Anxiety 26, 537–543. doi: 10.1002/da.20471, PMID: 19016462

[ref41] Li C. Hong Y. Yang X. Zeng X. Ocepek-Welikson K. Eimicke J. P.. (2023). The use of subjective cognitive complaints for detecting mild cognitive impairment in older adults across cultural and linguistic groups: a comparison of the cognitive function instrument to the Montreal cognitive assessment. Alzheimers Dement. 19, 1764–1774. doi: 10.1002/alz.12804, PMID: 36222321 PMC10090224

[ref42] Maffoni M. Pierobon A. Fundarò C. (2022). MASCoD-multidimensional assessment of subjective cognitive decline. Front. Psychol. 13:921062. doi: 10.3389/fpsyg.2022.921062, PMID: 36533024 PMC9748696

[ref43] Marra C. Piccininni C. Masone Iacobucci G. Caprara A. Gainotti G. Costantini E. M.. (2021). Semantic memory as an early cognitive marker of Alzheimer's disease: role of category and phonological verbal fluency tasks. J. Alzheimers Dis. 81, 619–627. doi: 10.3233/JAD-201452, PMID: 33814440

[ref44] Measso G. Zappalà G. Cavarzeran F. Crook T. H. Romani L. Pirozzolo F. J.. (1993). Raven's colored progressive matrices: a normative study of a random sample of healthy adults. Acta Neurol. Scand. 88, 70–74. doi: 10.1111/j.1600-0404.1993.tb04190.x, PMID: 8372633

[ref45] Molinuevo J. L. Rabin L. A. Amariglio R. Buckley R. Dubois B. Ellis K. A.. (2017). Implementation of subjective cognitive decline criteria in research studies. Alzheimers Dement. 13, 296–311. doi: 10.1016/j.jalz.2016.09.012, PMID: 27825022 PMC5344703

[ref9001] Mitchell A. J. Beaumont H. Ferguson D. Yadegarfar M. Stubbs B. (2014). Risk of dementia and mild cognitive impairment in older people with subjective memory complaints: meta-analysis. Acta psychiatrica Scandinavica 130, 439–451. doi: 10.1111/acps.12336, PMID: 25219393

[ref46] Numbers K. Lam B. C. P. Crawford J. D. Kochan N. A. Sachdev P. S. Brodaty H. (2021). Increased reporting of subjective cognitive complaints over time predicts cognitive decline and incident dementia. Int. J. Geriatr. Psychiatry 36, 1739–1747. doi: 10.1002/gps.5594, PMID: 34216392

[ref47] Orsini A. Grossi D. Capitani E. Laiacona M. Papagno C. Vallar G. (1987). Verbal and spatial immediate memory span: normative data from 1355 adults and 1112 children. Ital. J. Neurol. Sci. 8, 537–548. doi: 10.1007/BF02333660, PMID: 3429213

[ref48] Pestana P. C. Cardoso S. Guerreiro M. Maroco J. Jessen F. do Couto F. S.. (2024). Frequency, sociodemographic, and neuropsychological features of patients with subjective cognitive decline diagnosed using different neuropsychological criteria. Alzheimer's Res Ther 16:261. doi: 10.1186/s13195-024-01634-1, PMID: 39639343 PMC11619704

[ref49] Pigliautile M. Chiesi F. Stablum F. Rossetti S. Primi C. Chiloiro D.. (2019). Italian version and normative data of Addenbrooke's cognitive examination III. Int. Psychogeriatr. 31, 241–249. doi: 10.1017/S104161021800073X, PMID: 30021668

[ref50] Plummer F. Manea L. Trepel D. McMillan D. (2016). Screening for anxiety disorders with the GAD-7 and GAD-2: a systematic review and diagnostic metaanalysis. Gen. Hosp. Psychiatry 39, 24–31. doi: 10.1016/j.genhosppsych.2015.11.005, PMID: 26719105

[ref51] Qiu C. Fratiglioni L. (2015). A major role for cardiovascular burden in age-related cognitive decline. Nat. Rev. Cardiol. 12, 267–277. doi: 10.1038/nrcardio.2014.223, PMID: 25583619

[ref52] Rabin L. A. Smart C. M. Amariglio R. E. (2017). Subjective cognitive decline in preclinical Alzheimer's disease. Annu. Rev. Clin. Psychol. 13, 369–396. doi: 10.1146/annurev-clinpsy-032816-045136, PMID: 28482688

[ref53] Rabin L. A. Smart C. M. Crane P. K. Amariglio R. E. Berman L. M. Boada M.. (2015). Subjective cognitive decline in older adults: an overview of self-report measures used across 19 international research studies. J Alzheimer's Dis 48, S63–S86. doi: 10.3233/JAD-150154, PMID: 26402085 PMC4617342

[ref54] Reid M. Parkinson L. Gibson R. Schofield P. D'Este C. Attia J.. (2012). Memory complaint questionnaire performed poorly as screening tool: validation against psychometric tests and affective measures. J. Clin. Epidemiol. 65, 199–205. doi: 10.1016/j.jclinepi.2011.06.006, PMID: 21889305

[ref55] Röhr S. Pabst A. Riedel-Heller S. G. Jessen F. Turana Y. Handajani Y. S.. (2020). Estimating prevalence of subjective cognitive decline in and across international cohort studies of aging: a COSMIC study. Alzheimer's Res Ther 12:167. doi: 10.1186/s13195-020-00734-y, PMID: 33339532 PMC7749505

[ref56] Siciliano M. Chiorri C. Battini V. Sant'Elia V. Altieri M. Trojano L.. (2019). Regression-based normative data and equivalent scores for trail making test (TMT): an updated Italian normative study. Neurol. Sci. 40, 469–477. doi: 10.1007/s10072-018-3673-y, PMID: 30535956

[ref57] Singh-Manoux A. Dugravot A. Ankri J. Nabi H. Berr C. Goldberg M.. (2014). Subjective cognitive complaints and mortality: does the type of complaint matter? J. Psychiatr. Res. 48, 73–78. doi: 10.1016/j.jpsychires.2013.10.005, PMID: 24161314

[ref58] Smith G. Della Sala S. Logie R. H. Maylor E. A. (2000). Prospective and retrospective memory in normal ageing and dementia: a questionnaire study. Memory (Hove, England) 8, 311–321. doi: 10.1080/09658210050117735, PMID: 11045239

[ref9002] Slot R. E. R. Sikkes S. A. M. Berkhof J. Brodaty K. Buckley R. Cavedo E. (2019). Subjective cognitive decline and rates of incident Alzheimer’s disease and non-Alzheimer’s disease dementia. Alzheimer’s & dementia: the journal of the Alzheimer’s Association, 15, 465–476. doi: 10.1016/j.jalz.2018.10.003, PMID: 30555032 PMC6465066

[ref59] Spitzer R. L. Kroenke K. Williams J. B. (1999). Validation and utility of a self-report version of PRIME-MD: the PHQ primary care study. Primary care evaluation of mental disorders. Patient health questionnaire. JAMA 282, 1737–1744. doi: 10.1001/jama.282.18.1737, PMID: 10568646

[ref60] Spitzer R. L. Kroenke K. Williams J. B. Löwe B. (2006). A brief measure for assessing generalized anxiety disorder: the GAD-7. Arch. Intern. Med. 166, 1092–1097. doi: 10.1001/archinte.166.10.1092, PMID: 16717171

[ref61] Staples L. G. Dear B. F. Gandy M. Fogliati V. Fogliati R. Karin E.. (2019). Psychometric properties and clinical utility of brief measures of depression, anxiety, and general distress: the PHQ-2, GAD-2, and K-6. Gen. Hosp. Psychiatry 56, 13–18. doi: 10.1016/j.genhosppsych.2018.11.003, PMID: 30508772

[ref62] Tank R. Diaz A. Ashford M. T. Miller M. J. Eichenbaum J. Aaronson A.. (2024). Examining demographic factors, psychosocial wellbeing and cardiovascular health in subjective cognitive decline in the brain health registry cohort. J. Prev Alzheimers Dis. 11, 787–797. doi: 10.14283/jpad.2024.39, PMID: 38706295 PMC11061024

[ref63] The Jamovi Project. (2025). *Jamovi (version 2.6) [computer software]*. Available online at: https://www.jamovi.org.

[ref64] Tomata Y. Sugiyama K. Kaiho Y. Sugawara Y. Hozawa A. Tsuji I. (2017). Predictive ability of a simple subjective memory complaints scale for incident dementia: evaluation of Japan's national checklist, the "Kihon checklist". Geriatr Gerontol Int 17, 1300–1305. doi: 10.1111/ggi.12864, PMID: 27506749

[ref65] Vale F. A. C. Balieiro A. P. Silva-Filho J. H. (2012). Memory complaint scale (MCS). Proposed tool for active systematic search. Dement. Neuropsychol. 6, 212–218. doi: 10.1590/S1980-57642012DN06040004, PMID: 29213800 PMC5619332

[ref66] Walsh S. P. Raman R. Jones K. B. Aisen P. S. Alzheimer's Disease Cooperative Study Group (2006). ADCS prevention instrument project: the mail-in cognitive function screening instrument (MCFSI). Alzheimer Dis. Assoc. Disord. 20, S170–S178. doi: 10.1097/01.wad.0000213879.55547.57, PMID: 17135810

[ref67] Yates J. A. Clare L. Woods R. T. MRC CFAS (2017). Subjective memory complaints, mood and MCI: a follow-up study. Aging Ment. Health 21, 313–321. doi: 10.1080/13607863.2015.1081150, PMID: 26329364 PMC5351791

[ref68] Zhang H. Zhou Y. Ma J. Li Z. (2021). Understanding help-seeking decisions in people with subjective cognitive decline: a systematic review of qualitative studies. Geriatr. Nurs. (New York, N.Y.) 42, 1507–1516. doi: 10.1016/j.gerinurse.2021.10.013, PMID: 34735997

[ref69] Zlatar Z. Z. Moore R. C. Palmer B. W. Thompson W. K. Jeste D. V. (2014). Cognitive complaints correlate with depression rather than concurrent objective cognitive impairment in the successful aging evaluation baseline sample. J. Geriatr. Psychiatry Neurol. 27, 181–187. doi: 10.1177/0891988714524628, PMID: 24614203 PMC4255945

[ref70] Zlatar Z. Z. Muniz M. Galasko D. Salmon D. P. (2018). Subjective cognitive decline correlates with depression symptoms and not with concurrent objective cognition in a clinic-based sample of older adults. J. Gerontol. B Psychol. Sci. Soc. Sci. 73, 1198–1202. doi: 10.1093/geronb/gbw207, PMID: 28329816 PMC6146771

