# Supplementary material for: The interplay between cognitive and psychological factors in subjective cognitive decline: contribution to the validation of a new screening battery
Source: Front Psychol. 2025 Oct 9;16:1670551. doi: 10.3389/fpsyg.2025.1670551 (PMC12545073; doi:10.3389/fpsyg.2025.1670551)
Supplement: Supplementary file 1 [file Data_Sheet_1.docx]

Supplementary material in English language – MASCoD

**Multidimentional Assessment of Subjective Cognitive Decline (MASCoD) – first interview**

**Surname** ……………………………….……………………… **Name**………………………….………….. **Sex** ……… **Age** …….…………

**Compilation date** …………….…………………………… **Operator** ………………………………………. **Patient’s general practitioner** …………………………………………

| **CLINICAL AND SOCIO-ANAGRAPHIC INFORMATION** | | |
| --- | --- | --- |
| **Family status:**  1. Unmarried  2. Married/common law partner  3. Widower  4. Separated  5. Divorced | **Current occupation:**  1. Craftsman  2. Staying in parent/ Houseman or Housewife  3. Unemployed  4. Manager  5. Employee  6. Businessman  7. Teacher  8. Freelancer  9. Worker  10. Disable  11. Retired  12. Other | **Whom do you live with**:  1. By myself  2. Husband/wife/partner  3. Son/Daughter  4. Partner and children  5. Parents  6. Other family members  7. Others (no family members) |
| **Education:**  1. None  2. Less than 5 years  3. 6 – 8 years of school  4. 9 – 13 years of school  5. More than 14 years or Bachelor Degree |  |  |
|  |  | **Primary caregiver:**  1. Husband/wife/partner  2. Son/Daughter  3. Parents  4. Other family members  5. Other members, not family (caregiver)  6. Nobody |
| **BMI …………………**  Weight: (Kg)…………  Height: (cm)………… | **Anamnesis risk factors**  *Please check more than one box, if necessary*  1. Smoking  2. Dyslipidaemia  3. Hypertension  4. Diabetes  5. Overweight  6. Alcohol: 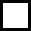 no 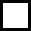 use 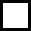 actual or past abuse  7. Addictions to drugs/psychostimulant drugs  8. Familiarity with other pathologies __________________________________________ | |
| **Actual smoking:**  1. Yes  2. No  3. Yes, in the past |  |  |

**(MASCoD) – first interview**

**SECTION A – Risk factors**

|  | **YES** | **NO** |
| --- | --- | --- |
| Onset of SCD within the last 5 years | 1 | 0 |
| Age at onset of SCD ≥ 60 | 1 | 0 |
| Concerns and worries associated with SCD | 1 | 0 |
| Persistence of SCD over time | 1 | 0 |
| Medical help seeking | 1 | 0 |
| Confirmation of cognitive decline by an informant | 1 | 0 |

|  | **YES** | **NO** |
| --- | --- | --- |
| Familiarity with movement disorders | 1 | 0 |
| Familiarity with cognitive disorders | 1 | 0 |
| Cerebral/neurological comorbidities | 1 | 0 |
| Other comorbidities (please, specify):  ________________________________ | 1 | 0 |

**SECTION B - MAC-Q** *(Crook et al., 1992, modified)*

How often: 1, 2 (often), 3(very often), 4(almost always)

Thinking about the **last period/last few months**:

| *Have you been able of:* | **YES** | **NO** |
| --- | --- | --- |
| Remembering a person just introduced to you (names, faces, ...)? If No, how often? 1 – 2 – 3 – 4 | 0 | 1 |
| Recalling password or other access codes that you use on a daily or weekly basis? If No, how often? 1 – 2 – 3 – 4 | 0 | 1 |
| Recalling where you have put objects (glasses, keys, …) If No, how often? 1 – 2 – 3 – 4 | 0 | 1 |
| Remembering specific facts from a newspaper, TV, or people conversation? If No, how often? 1 – 2 – 3 – 4 | 0 | 1 |
| Remembering the item(s) you intended to buy when you arrive at the grocery store or pharmacy? If No, how often? 1 – 2 – 3 – 4 | 0 | 1 |
| *In general, do you think your memory is worse than in the past?* | 1 | 0 |

| *Is it happen to you to:* | **YES** | **NO** |
| --- | --- | --- |
| Have moments of confusion/disorientation/short black-out? If Yes, how often? 1 – 2 – 3 – 4 | 1 | 0 |
| Have more difficulty in making decisions or planning than in the past? If Yes, how often? 1 – 2 – 3 – 4 | 1 | 0 |
| Forgetting important appointments more frequently than in the past? If Yes, how often? 1 – 2 – 3 – 4 | 1 | 0 |
| Have more difficulty following movies/news/conversations/readings than in the past? If Yes, how often? 1 – 2 – 3 – 4 | 1 | 0 |
| Have more problems of maintaining attention than in the past? If Yes, how often? 1 – 2 – 3 – 4 | 1 | 0 |

**SECTION C - GAD-2 & PHQ-2** *(Giuliani et al., 2020; Spitzer et al., 2006; 1999)*

| *Over the last 2 weeks, how often have you been bothered by the following problems?* | Not at all | Several  days | More than half the days | Nearly every day |
| --- | --- | --- | --- | --- |
| GAD-2 Feeling nervous, anxious, or on edge | 0 | 1 | 2 | 3 |
| GAD-2 Not being able to stop or control worrying | 0 | 1 | 2 | 3 |
| PHQ-2 Little interest or pleasure in doing things | 0 | 1 | 2 | 3 |
| PHQ-2 Feeling down, depressed or hopeless | 0 | 1 | 2 | 3 |
| *GAD-2 Total (cutoff >3)…. PHQ-2 Total (cutoff >2)…. Distress (cutoff moderate >6)….* | | | | |

| **SECTION C – STRESS** *Have any stressful life events (positive and/or negative) happened in the past year? Specify ….* | YES (1) | NO (0) |
| --- | --- | --- |

**Total Section A + B (0-21) _____________ Follow up suggested: 6 months 12 months extensive neuropsychological evaluation**

……………………………………………………………………………………………………………………………………………………………………………………………………

14-21: High risk: **Extensive neuropsychological evaluation** (and possible a psychological intervention for mood disorders if anxiety and depression are ABOVE the cut-off)

08-13: Medium risk: If anxiety and depression are **ABOVE the cut-off**, proceed with a **psychological intervention for mood disorders and follow-up at six months.**

*(If at six-month follow-up the score remains essentially unchanged [medium risk], continue with extensive neuropsychological evaluation)*

If anxiety and depression are **BELOW the cut-off**, proceed with an **extensive neuropsychological assessment** (If no deficits emerge, follow-up at 6 months).

0-07: Low risk: **1-year follow-up** (and a possible psychological intervention for mood disorders if anxiety and depression are ABOVE the cut-off)

**Multidimentional Assessment of Subjective Cognitive Decline (MASCoD) – follow up**

**Surname** ……………………………….……………………… **Name**………………………….………….. **Sex** ……… **Age** …….…………

**Compilation date** …………….…………………………… **Operator** ………………………………………. **Patient’s general practitioner** …………………………………………

**Previous risk level** (date of the first assessment ………………………………): low medium high

| **CLINICAL AND SOCIO-ANAGRAPHIC INFORMATION** | | |
| --- | --- | --- |
| **Family status:**  1. Unmarried  2. Married/common law partner  3. Widower  4. Separated  5. Divorced | **Current occupation:**  1. Craftsman  2. Staying in parent/Houseman or Housewife  3. Unemployed  4. Manager  5. Employee  6. Businessman  7. Teacher  8. Freelancer  9. Worker  10. Disable  11. Retired  12. Other | **Whom do you live with**:  1. By myself  2. Husband/wife/partner  3. Son/Daughter  4. Partner and children  5. Parents  6. Other family members  7. Others (no family members) |
| **Education:**  1. None  2. Less than 5 years  3. 6 – 8 years of school  4. 9 – 13 years of school  5. More than 14 years or Bachelor Degree |  |  |
|  |  | **Primary caregiver:**  1. Husband/wife/partner  2. Son/Daughter  3. Parents  4. Other family members  5. Other members, not family (caregiver)  6. Nobody |
| **BMI …………………**  **Weight**: (Kg)…………  **Height**: (cm)………… | **Anamnesis risk factors**  *Please check more than one box, if necessary*  1. Smoking  2. Dyslipidaemia  3. Hypertension  4. Diabetes  5. Overweight  6. Alcohol abuse: 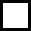 no 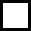 use 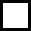 actual or past abuse  7. Addictions to drugs/psychostimulant drugs  8. Familiarity with other pathologies __________________________________________ | |
| **Actual smoking:**  1. Yes  2. No  3. Yes, in the past |  |  |

**MASCoD – follow up**

**SECTION A – Risk factors (OCCURRED AFTER THE LAST ASSESSMENT)**

|  | **SI** | **NO** |
| --- | --- | --- |
| Risk factors previously unveiled in Section A (**n° 0-10**) | n…. |  |
| Other cerebral/neurological comorbidities **occurred after the last assessment** | 1 | 0 |
| Other comorbidities **occurred after the last assessment** | 1 | 0 |

**SECTION B - MAC-Q** *(Crook et al., 1992, modified)*

How often: 1, 2 (often), 3(very often), 4(almost always)

Thinking about the **last period/last few months**:

| *Have you been able of:* If No, how often? 1 – 2 – 3 – 4 | **SI** | **NO** |
| --- | --- | --- |
| Remembering a person just introduced to you (names, faces, ...)? If No, how often? 1 – 2 – 3 – 4 | 0 | 1 |
| Recalling password or other access codes that you use on a daily or weekly basis? If No, how often? 1 – 2 – 3 – 4 | 0 | 1 |
| Recalling where you have put objects (glasses, keys, …) If No, how often? 1 – 2 – 3 – 4 | 0 | 1 |
| Remembering specific facts from a newspaper, TV, or people conversation? If No, how often? 1 – 2 – 3 – 4 | 0 | 1 |
| Remembering the item(s) you intended to buy when you arrive at the grocery store or pharmacy? If No, how often? 1 – 2 – 3 – 4 | 0 | 1 |
| *In general, do you think your memory is worse than in the past?* | 1 | 0 |

| *Is it happen to you to:* | **SI** | **NO** |
| --- | --- | --- |
| Have moments of confusion/disorientation/short black-out? If Yes, how often? 1 – 2 – 3 – 4 | 1 | 0 |
| Have more difficulty in making decisions or planning than in the past? If Yes, how often? 1 – 2 – 3 – 4 | 1 | 0 |
| Forgetting important appointments more frequently than in the past? If Yes, how often? 1 – 2 – 3 – 4 | 1 | 0 |
| Have more difficulty following movies/news/conversations/readings than in the past? If Yes, how often? 1 – 2 – 3 – 4 | 1 | 0 |
| Have more problems of maintaining attention than in the past? If Yes, how often? 1 – 2 – 3 – 4 | 1 | 0 |

**SECTION C - GAD-2 & PHQ-2** *(Giuliani et al., 2020; Spitzer et al., 2006; 1999)*

| *Over the last 2 weeks, how often have you been bothered by the following problems?* | Not at all | Several  days | More than half the days | Nearly every day |
| --- | --- | --- | --- | --- |
| GAD-2 Feeling nervous, anxious, or on edge | 0 | 1 | 2 | 3 |
| GAD-2 Not being able to stop or control worrying | 0 | 1 | 2 | 3 |
| PHQ-2 Little interest or pleasure in doing things | 0 | 1 | 2 | 3 |
| PHQ-2 Feeling down, depressed or hopeless | 0 | 1 | 2 | 3 |
| *GAD-2 Total (cutoff >3)…. PHQ-2 Total (cutoff >2)…. Distress (cutoff moderate >6)….* | | | | |

| **SECTION C STRESS –** *Have any stressful life events (positive and/or negative) happened in the past year?* *Specify ….* | Si (1) | No (0) |
| --- | --- | --- |

**Total Section A + B (0-23) _____________ Follow up suggested: 6 months 12 months extensive neuropsychological evaluation**

……………………………………………………………………………………………………………………………………………………………………………………………

16-21: High risk: **Extensive neuropsychological evaluation** (and a possible psychological intervention for mood disorders if anxiety and depression are ABOVE the cut-off)

10-15: Medium risk: If at the six-month follow-up the score remains substantially **unchanged** [medium risk], continue with the **extensive neuropsychological evaluation and further follow-up.**

If anxiety and depression are **ABOVE the cut-off**, proceed with a **psychological intervention and/or referral to another specialist to evaluate the drug therapy.**

0-09: Low risk: **1-year follow-up** (and a possible psychological intervention for mood disorders if anxiety and depression are ABOVE the cut-off)

Supplementary material in Italian language – MASCoD

**Multidimentional Assessment of Subjective Cognitive Decline (MASCoD) – prima visita**

**Cognome** ……………………………….………………………… **Nome**………………………….………….. **Sesso** ……… **Età**  …….…………

**Data di compilazione**…………….…………………………… **Compilatore: operatore sanitario** ………………………………………. M**edico di riferimento** …………………………………………

| **DATI CLINICI E SOCIO-ANAGRAFICI** | | |
| --- | --- | --- |
| **1. Stato civile**:  1. Libero  2. Coniugato-convivente  3. Vedovo  4. Separato  5. Divorziato | **3. Attività lavorativa**:  1. Artigiano/commerciante  2. Casalinga  3. Disoccupato  4. Dirigente  5. Impiegato  6. Imprenditore  7. Insegnante  8. Libero professionista  9. Operaio  10. Invalido  11. Pensionato  12. Altro | **4. Con chi vive**: Barrare anche più di una risposta  1. Solo  2. Con coniuge-convivente  3. Con figli  4. Con coniuge e figli  5. Con parenti (non coniuge o figli)  6. Con altre persone (no familiari) |
| **2. Titolo di studio**:  1. Nessuno  2. Licenza elementare  3. Licenza scuola media inferiore  4. Diploma scuola media superiore  5. Diploma universitario  6. Laurea |  |  |
|  |  | **5. Principale persona di riferimento per la cura**  1. Partner  2. Figlio/i  3. Assistente alla persona (es. badante, colf…)  4. Amico/vicino di casa o altra persona della rete sociale  5. Nessuno |
| **6. BMI**  **peso**: (Kg) ……………..  **altezza**: (cm) ………… | **8. Anamnesi fattori di rischio** *(Barrare anche più di una risposta):*  1. Dislipidemia  2. Ipertensione arteriosa  3. Diabete  4. Iperuricemia  5. Sovrappeso  6. Alcool: 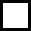 no 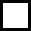 uso 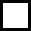 abuso attuale o *potus* in anamnesi  7. Dipendenza da sostanze/farmaci psicostimolanti | |
| **7. Fumo attuale**  1. Sì  2. No  3. Si in passato |  |  |

**MASCoD – prima visita**

**SEZIONE A - Fattori di rischio**

|  | **SI** | **NO** |
| --- | --- | --- |
| Insorgenza di SCD negli ultimi 5 anni | 1 | 0 |
| Insorgenza di SCD > 60 anni | 1 | 0 |
| Preoccupazione associata a SCD | 1 | 0 |
| Persistenza della SCD nel tempo | 1 | 0 |
| Ricerca di assistenza medica | 1 | 0 |
| Conferma di SCD da parte di un osservatore esterno | 1 | 0 |

|  | **SI** | **NO** |
| --- | --- | --- |
| Familiarità per disturbi del movimento | 1 | 0 |
| Familiarità per disturbi cognitivi | 1 | 0 |
| Comorbidità neurologiche/cerebrali | 1 | 0 |
| Altre comorbidità organiche (specificare):  ________________________________ | 1 | 0 |

**SEZIONE B – Manifestazioni cognitive soggettive**

Quanto spesso: 1(raramente), 2 (spesso), 3(molto spesso), 4(quasi sempre)

Pensando **all’ultimo periodo/ultimi mesi**:

| *È riuscito a:* | **SI** | **NO** |
| --- | --- | --- |
| Ricordare le persone da poco presentate (nomi, viso, ...)? Se NO, quanto spesso? 1 – 2 – 3 – 4 | 0 | 1 |
| Ricordare i codici di accesso (PIN, password ….) che usa ogni giorno o almeno una volta a settimana? Se NO, quanto spesso? 1 – 2 – 3 – 4 | 0 | 1 |
| Ricordare dove si appoggiano gli oggetti (chiavi, occhiali…)? Se NO, quanto spesso? 1 – 2 – 3 – 4 | 0 | 1 |
| Ricordare eventi (fatti) precisi che ho appena letto nel giornale o sentito alla TV o in una conversazione? Se NO, quanto spesso? 1 – 2 – 3 – 4 | 0 | 1 |
| Ricordare le cose da comperare quando entra in un negozio o in farmacia? Se NO, quanto spesso? 1 – 2 – 3 – 4 | 0 | 1 |
| *Complessivamente la sua memoria di adesso è peggiorata rispetto a quella del passato* | 1 | 0 |

| *Le è capitato di:* | **SI** | **NO** |
| --- | --- | --- |
| Avere momenti di confusione/disorientamento/ piccoli black-out? Se SI, quanto spesso? 1 – 2 – 3 – 4 | 1 | 0 |
| Avere maggiori difficoltà a prendere decisioni o nel pianificare rispetto al passato? Se SI, quanto spesso? 1 – 2 – 3 – 4 | 1 | 0 |
| Dimenticare appuntamenti importanti più frequentemente rispetto al passato? Se SI, quanto spesso? 1 – 2 – 3 – 4 | 1 | 0 |
| Avere maggiori difficoltà a seguire film/telegiornali/conversazioni/letture rispetto al passato? Se SI, quanto spesso? 1 – 2 – 3 – 4 | 1 | 0 |
| Avere maggiori momenti di distrazione rispetto al passato? Se SI, quanto spesso? 1 – 2 – 3 – 4 | 1 | 0 |

**SEZIONE C – Manifestazioni psicologiche**

| *Nelle ultime 2 settimane, con quale frequenza le ha dato fastidio ciascuno dei seguenti problemi?* | Mai | Alcuni giorni | Per oltre la metà dei giorni | Quasi ogni  giorno |
| --- | --- | --- | --- | --- |
| GAD-2 Sentirsi nervoso/a, ansioso/a o teso/a | 0 | 1 | 2 | 3 |
| GAD-2 Non riuscire a smettere di preoccuparsi o a tenere sotto controllo le preoccupazioni | 0 | 1 | 2 | 3 |
| PHQ-2 Scarso interesse o piacere nel fare le cose | 0 | 1 | 2 | 3 |
| PHQ-2 Sentirsi giù, triste o disperato/a | 0 | 1 | 2 | 3 |
| *GAD-2 Totale (cutoff >3) …. PHQ-2 Totale (cutoff >2) …. Distress Totale (cutoff moderate >6) ….* | | | | |

| **SEZIONE C – Stress**  *Nell’ultimo anno sono successi eventi di vita stressanti (positivi e/o negativi)? Specificare …….* | SI (1) | NO (0) |
| --- | --- | --- |

**Totale SEZ A + B (0-21) _____________ Controllo consigliato: 6 mesi 12 mesi Valutazione neuropsicologica approfondita**

……………………………………………………………………………………………………………………………………………………………………………………………………

14-21: Rischio alto: **Valutazione neuropsicologica** (ed eventuale supporto diagnostico-clinico per disturbi dell’umore se ansia, depressione e/o distress sono presenti, cioè SOPRA il cutoff)

8-13: Rischio medio: Se ansia, depressione e/o distress presenti (**SOPRA cutoff**), procedere con **approfondimento sui disturbi dell’umore e follow-up a sei mesi.**

*(Se nel follow-up a sei mesi il punteggio il punteggio rimane sostanzialmente invariato [rischio medio],* ***proseguire con valutazione neuropsicologica****).*

Se ansia, depressione e/o distress assenti (**SOTTO cutoff**), procedere con **valutazione neuropsicologica e follow-up a 6 mesi**.

0-7: Rischio basso: **Follow-up a 1 anno** (ed eventuale supporto diagnostico-clinico per disturbi dell’umore se ansia e depressione sono presenti, cioè SOPRA il cutoff)

**Multidimentional Assessment of Subjective Cognitive Decline (MASCoD) –** **follow up**

**Cognome** ……………………………….………………………… **Nome**………………………….………….. **Età**  …….…………

**Data di compilazione**…………….…………………………… **Compilatore: operatore sanitario** ………………………………………. M**edico di riferimento** …………………………………………

**Livello di rischio precedentemente rilevato** (in data ………………………………..): basso medio alto

| **DATI CLINICI E SOCIO-ANAGRAFICI** | | |
| --- | --- | --- |
| **1. Stato civile**:  1. Libero  2. Coniugato-convivente  3. Vedovo  4. Separato  5. Divorziato | **3. Attività lavorativa**:  1. Artigiano/commerciante  2. Casalinga  3. Disoccupato  4. Dirigente  5. Impiegato  6. Imprenditore  7. Insegnante  8. Libero professionista  9. Operaio  10. Invalido  11. Pensionato  12. Altro | **4. Con chi vive**: Barrare anche più di una risposta  1. Solo  2. Con coniuge-convivente  3. Con figli  4. Con coniuge e figli  5. Con parenti (non coniuge o figli)  6. Con altre persone (no familiari) |
| **2. Titolo di studio**:  1. Nessuno  2. Licenza elementare  3. Licenza scuola media inferiore  4. Diploma scuola media superiore  5. Diploma universitario  6. Laurea |  |  |
|  |  | **5. Principale persona di riferimento per la cura**  1. Partner  2. Figlio/i  3. Assistente alla persona (es. badante, colf…)  4. Amico/vicino di casa o altra persona della rete sociale  5. Nessuno |
| **6. BMI**  **peso**: (Kg) ……………..  **altezza**: (cm) ………… | **8. Anamnesi fattori di rischio** *(Barrare anche più di una risposta):*  1. Dislipidemia  2. Ipertensione arteriosa  3. Diabete  4. Iperuricemia  5. Sovrappeso  6. Alcool: 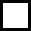 no 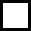 uso 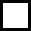 abuso attuale o *potus* in anamnesi  7. Dipendenza da sostanze/farmaci psicostimolanti | |
| **7. Fumo attuale**  1. Sì  2. No  3. Si in passato |  |  |

**MASCoD - follow up**

**SEZIONE A - Fattori di rischio SOPRAGGIUNTI DOPO L’ULTIMO CONTROLLO**

|  | **SI** | **NO** |
| --- | --- | --- |
| Fattori di rischio precedentemente rilevati nella sezione A (n° 0-10) | n…. |  |
| Comorbidità neurologiche/cerebrali **sopraggiunte dalla precedente valutazione** | 1 | 0 |
| Altre comorbidità organiche **sopraggiunte dalla precedente valutazione** | 1 | 0 |

**SEZIONE B - Manifestazioni cognitive soggettive**

Quanto spesso: 1(raramente), 2 (spesso), 3(molto spesso), 4(quasi sempre)

Pensando **all’ultimo periodo/ultimi mesi**:

| *É riuscito a:* | **SI** | **NO** |
| --- | --- | --- |
| Ricordare le persone da poco presentate (nomi, viso, ...)? Se NO, quanto spesso? 1 – 2 – 3 – 4 | 0 | 1 |
| Ricordare i codici di accesso (PIN, password ….) che usa ogni giorno o almeno una volta a settimana? Se NO, quanto spesso? 1 – 2 – 3 – 4 | 0 | 1 |
| Ricordare dove si appoggiano gli oggetti (chiavi, occhiali…)? Se NO, quanto spesso? 1 – 2 – 3 – 4 | 0 | 1 |
| Ricordare eventi (fatti) precisi che ho appena letto nel giornale o sentito alla TV o in una conversazione? Se NO, quanto spesso? 1 – 2 – 3 – 4 | 0 | 1 |
| Ricordare le cose da comperare quando entra in un negozio o in farmacia? Se NO, quanto spesso? 1 – 2 – 3 – 4 | 0 | 1 |
| *Complessivamente la sua memoria di adesso è peggiorata rispetto a quella del passato?* | 1 | 0 |

| *Le è capitato di:* | **SI** | **NO** |
| --- | --- | --- |
| Avere momenti di confusione/disorientamento/ piccoli black-out? Se SI, quanto spesso? 1 – 2 – 3 – 4 | 1 | 0 |
| Avere maggiori difficoltà a prendere decisioni o nel pianificare rispetto al passato? Se SI, quanto spesso? 1 – 2 – 3 – 4 | 1 | 0 |
| Dimenticare appuntamenti importanti più frequentemente rispetto al passato? Se SI, quanto spesso? 1 – 2 – 3 – 4 | 1 | 0 |
| Avere maggiori difficoltà a seguire film/telegiornali/conversazioni/letture rispetto al passato? Se SI, quanto spesso? 1 – 2 – 3 – 4 | 1 | 0 |
| Avere maggiori momenti di distrazione rispetto al passato? Se SI, quanto spesso? 1 – 2 – 3 – 4 | 1 | 0 |

**SEZIONE C - Manifestazioni psicologiche**

| *Nelle ultime 2 settimane, con quale frequenza le ha dato fastidio ciascuno dei seguenti problemi?* | Mai | Alcuni giorni | Per oltre la metà dei giorni | Quasi ogni  giorno |
| --- | --- | --- | --- | --- |
| GAD-2 Sentirsi nervoso/a, ansioso/a o teso/a | 0 | 1 | 2 | 3 |
| GAD-2 Non riuscire a smettere di preoccuparsi o a tenere sotto controllo le preoccupazioni | 0 | 1 | 2 | 3 |
| PHQ-2 Scarso interesse o piacere nel fare le cose | 0 | 1 | 2 | 3 |
| PHQ-2 Sentirsi giù, triste o disperato/a | 0 | 1 | 2 | 3 |
| *GAD-2 Totale (cutoff >3) …. PHQ-2 Totale (cutoff >2) …. Distress Totale (cutoff moderate >6) ….* | | | | |

| **SEZIONE C – Stress** *Nell’ultimo anno sono successi eventi di vita stressanti (positivi e/o negativi)? Specificare …..* | SI (1) | NO (0) |
| --- | --- | --- |

**Totale SEZ A + B (0-23) _____________ Controllo consigliato: 6 mesi 12 mesi Valutazione neuropsicologica approfondita**

……………………………………………………………………………………………………………………………………………………………………………………………

16-23: Rischio alto: **Valutazione neuropsicologica** (ed eventuale supporto diagnostico-clinico per disturbi dell’umore se ansia, depressione e/o distress sono presenti, cioè SOPRA il cutoff)

10-15: Se al follow-up di sei mesi il punteggio rimane sostanzialmente **invariato** [rischio medio], continuare con la **valutazione neuropsicologica approfondita e un ulteriore follow-up**.

Se l'ansia e la depressione sono **SOPRA il cut-off**, procedere con un **intervento psicologico e/o rinviare a un altro specialista per valutare la terapia farmacologica**.

0-9: Rischio basso: **Follow-up a 1 anno** (ed eventuale supporto diagnostico-clinico per disturbi dell’umore se ansia, depressione e/o distress sono presenti, cioè SOPRA il cutoff)
