# Supplementary material for: The interplay between cognitive and psychological factors in subjective cognitive decline: contribution to the validation of a new screening battery
Source: Front Psychol. 2025 Oct 9;16:1670551. doi: 10.3389/fpsyg.2025.1670551 (PMC12545073; doi:10.3389/fpsyg.2025.1670551)
Supplement: Supplementary file 2 [file Supplementary_file_1.docx]

| **APPENDIX A - Correlation Matrix** | | | | |
| --- | --- | --- | --- | --- |
|  |  | MASCoD Section A | MASCoD Section B | MASCoD Section A+B |
| MASCoD Section A | Pearson's r | — |  |  |
|  | df | — |  |  |
|  | p-value | — |  |  |
| MASCoD Section B | Pearson's r | 0.224 | — |  |
|  | df | 55 | — |  |
|  | p-value | 0.094 | — |  |
| MASCoD Section A+B | Pearson's r | 0.580*** | 0.923*** | — |
|  | df | 55 | 55 | — |
|  | p-value | <.001 | <.001 | — |
| MMSE | Pearson's r | -0.032 | 0.084 | 0.055 |
|  | df | 55 | 55 | 55 |
|  | p-value | 0.812 | 0.534 | 0.686 |
| ACE-III | Pearson's r | 0.062 | -0.342* | -0.264 |
|  | df | 48 | 48 | 48 |
|  | p-value | 0.668 | 0.015 | 0.064 |
| FAB | Pearson's r | 0.141 | 0.246 | 0.254 |
|  | df | 55 | 55 | 55 |
|  | p-value | 0.294 | 0.065 | 0.056 |
| Rey Figure Copy | Pearson's r | 0.020 | 0.153 | 0.132 |
|  | df | 55 | 55 | 55 |
|  | p-value | 0.883 | 0.254 | 0.327 |
| Rey Figure Recall | Pearson's r | 0.038 | -0.071 | -0.054 |
|  | df | 55 | 55 | 55 |
|  | p-value | 0.778 | 0.599 | 0.691 |
| Digit Span Forward | Pearson's r | -0.011 | 0.005 | -0.004 |
|  | df | 55 | 55 | 55 |
|  | p-value | 0.936 | 0.969 | 0.978 |
| Digit Span Backward | Pearson's r | -0.009 | 0.007 | 0.001 |
|  | df | 55 | 55 | 55 |
|  | p-value | 0.947 | 0.961 | 0.996 |
| Phonological Fluency | Pearson's r | 0.103 | 0.124 | 0.144 |
|  | df | 55 | 55 | 55 |
|  | p-value | 0.447 | 0.362 | 0.286 |
| Semantic Fluency | Pearson's r | -0.070 | -0.089 | -0.110 |
|  | df | 55 | 55 | 55 |
|  | p-value | 0.607 | 0.508 | 0.415 |
| Corsi Forward | Pearson's r | 0.069 | 0.010 | 0.024 |
|  | df | 55 | 55 | 55 |
|  | p-value | 0.612 | 0.944 | 0.858 |
| Corsi Backward | Pearson's r | 0.053 | -0.080 | -0.055 |
|  | df | 55 | 55 | 55 |
|  | p-value | 0.693 | 0.553 | 0.687 |
| TMT A | Pearson's r | -0.320* | 0.193 | 0.038 |
|  | df | 55 | 55 | 55 |
|  | p-value | 0.015 | 0.151 | 0.779 |
| TMT B | Pearson's r | -0.097 | -0.045 | -0.074 |
|  | df | 53 | 53 | 53 |
|  | p-value | 0.479 | 0.744 | 0.590 |
| CDT | Pearson's r | -0.123 | 0.008 | -0.043 |
|  | df | 55 | 55 | 55 |
|  | p-value | 0.362 | 0.955 | 0.750 |
| 15-word Immediate Recall | Pearson's r | 0.137 | -0.033 | 0.017 |
|  | df | 55 | 55 | 55 |
|  | p-value | 0.852 | 0.757 | 0.806 |
| 15-word Delayed Recall | Pearson's r | 0.025 | -0.042 | -0.053 |
|  | df | 53 | 53 | 53 |
|  | p-value | 0.505 | 0.084 | 0.092 |
| Stroop Time | Pearson's r | -0.092 | -0.235 | -0.230 |
|  | df | 54 | 54 | 54 |
|  | p-value | 0.505 | 0.084 | 0.092 |
| Stroop Errors | Pearson's r | -0.029 | -0.222 | -0.199 |
|  | df | 54 | 54 | 54 |
|  | p-value | 0.830 | 0.100 | 0.142 |
| Raven | Pearson's r | 0.088 | -0.044 | -0.017 |
|  | df | 55 | 55 | 55 |
|  | p-value | 0.514 | 0.744 | 0.900 |
| PHQ-9 | Pearson's r | 0.327* | 0.213 | 0.299* |
|  | df | 55 | 55 | 55 |
|  | p-value | 0.013 | 0.112 | 0.024 |
| GAD-7 | Pearson's r | 0.236 | -0.051 | 0.046 |
|  | df | 55 | 55 | 55 |
|  | p-value | 0.077 | 0.705 | 0.735 |
| *Note.* * p < .05, ** p < .01, *** p < .001 | | | | |
