# Supplementary material for: The interplay between cognitive and psychological factors in subjective cognitive decline: contribution to the validation of a new screening battery
Source: Front Psychol. 2025 Oct 9;16:1670551. doi: 10.3389/fpsyg.2025.1670551 (PMC12545073; doi:10.3389/fpsyg.2025.1670551)
Supplement: Supplementary file 3 [file Supplementary_file_2.docx]

| **APPENDIX B - Correlation Matrix** | | |
| --- | --- | --- |
|  |  | CFI self-report |
| CFI self-report | Pearson's r | — |
|  | df | — |
|  | p-value | — |
| MMSE | Pearson's r | 0.146 |
|  | df | 47 |
|  | p-value | 0.316 |
| ACE-III | Pearson's r | 0.015 |
|  | df | 40 |
|  | p-value | 0.926 |
| FAB | Pearson's r | 0.031 |
|  | df | 47 |
|  | p-value | 0.835 |
| Rey Figure Copy | Pearson's r | -0.116 |
|  | df | 47 |
|  | p-value | 0.429 |
| Rey Figure Recall | Pearson's r | 0.020 |
|  | df | 47 |
|  | p-value | 0.890 |
| Digit Span Forward | Pearson's r | -0.097 |
|  | df | 47 |
|  | p-value | 0.507 |
| Digit Span Backward | Pearson's r | -0.069 |
|  | df | 47 |
|  | p-value | 0.639 |
| Phonological Fluency | Pearson's r | 0.072 |
|  | df | 47 |
|  | p-value | 0.622 |
| Semantic Fluency | Pearson's r | -0.133 |
|  | df | 47 |
|  | p-value | 0.361 |
| Corsi Forward | Pearson's r | -0.058 |
|  | df | 47 |
|  | p-value | 0.692 |
| Corsi Backward | Pearson's r | -0.293* |
|  | df | 47 |
|  | p-value | 0.041 |
| TMT A | Pearson's r | 0.018 |
|  | df | 47 |
|  | p-value | 0.900 |
| TMT B | Pearson's r | -0.078 |
|  | df | 45 |
|  | p-value | 0.600 |
| CDT | Pearson's r | -0.100 |
|  | df | 47 |
|  | p-value | 0.495 |
| 15-word Immediate Recall | Pearson's r | 0.042 |
|  | df | 47 |
|  | p-value | 0.772 |
| 15-word Delayed Recall | Pearson's r | 0.095 |
|  | df | 47 |
|  | p-value | 0.516 |
| Stroop Time | Pearson's r | -0.034 |
|  | df | 45 |
|  | p-value | 0.822 |
| Raven | Pearson's r | -0.155 |
|  | df | 47 |
|  | p-value | 0.287 |
| PHQ-9 | Pearson's r | 0.328* |
|  | df | 47 |
|  | p-value | 0.022 |
| GAD-7 | Pearson's r | 0.026 |
|  | df | 47 |
|  | p-value | 0.860 |
| *Note.* * p < .05, ** p < .01, *** p < .001 | | |
